# Dual P-Glycoprotein and CA XII Inhibitors: A New Strategy to Reverse the P-gp Mediated Multidrug Resistance (MDR) in Cancer Cells †

**DOI:** 10.3390/molecules25071748

**Published:** 2020-04-10

**Authors:** Elisabetta Teodori, Laura Braconi, Silvia Bua, Andrea Lapucci, Gianluca Bartolucci, Dina Manetti, Maria Novella Romanelli, Silvia Dei, Claudiu T. Supuran, Marcella Coronnello

**Affiliations:** 1 Department of Neuroscience, Psychology, Drug Research and Child’s Health–Section of Pharmaceutical and Nutraceutical Sciences, University of Florence, via Ugo Schiff 6, 50019 Sesto Fiorentino (FI), Italy; elisabetta.teodori@unifi.it (E.T.); laura.braconi@unifi.it (L.B.); silvia.bua@unifi.it (S.B.); gianluca.bartolucci@unifi.it (G.B.); dina.manetti@unifi.it (D.M.); novella.romanelli@unifi.it (M.N.R.); claudiu.supuran@unifi.it (C.T.S.); 2 Department of Health Sciences—Section of Clinical Pharmacology and Oncology, University of Florence, Viale Pieraccini 6, 50139 Firenze, Italy; andrea.lapucci@unifi.it (A.L.); marcella.coronnello@unifi.it (M.C.)

**Keywords:** MDR reversers, P-gp modulators, CA XII inhibitors, K562/DOX, LoVo/DOX, hybrid compounds, multitarget ligands, dual P-gp/CA XII inhibitory activity, selective chemosensitizers

## Abstract

A new series of *N,N*-bis(alkanol)amine aryl diesters was synthesized and studied as dual P-glycoprotein (P-gp) and carbonic anhydrase XII inhibitors (CA XII). These hybrids should be able to synergistically overcome P-gp mediated multidrug resistance (MDR) in cancer cells. It was reported that the efflux activity of P-gp could be modulated by CA XII, as the pH reduction caused by CA XII inhibition produces a significant decrease in P-gp ATPase activity. The new compounds reported here feature both P-gp and CA XII binding moieties. These hybrids contain a *N,N*-bis(alkanol)amine diester scaffold found in P-glycoprotein ligands and a coumarin or benzene sulfonamide moiety to target CA XII. Many compounds displayed a dual activity against P-gp and CA XII being active in the Rhd 123 uptake test on K562/DOX cells and in the hCA XII inhibition test. On LoVo/DOX cells, that overexpress both P-gp and CA XII, some coumarin derivatives showed a high MDR reversal effect in Rhd 123 uptake and doxorubicin cytotoxicity enhancement tests. In particular, compounds **7** and **8** showed higher activity than verapamil and were more potent on LoVo/DOX than on K562/DOX cells overexpressing only P-gp. They can be considered as valuable candidates for selective P-gp/CA XII inhibition in MDR cancer cells.

## 1. Introduction

The resistance of many tumor cells to structurally and mechanistically unrelated antineoplastic drugs is the main failure reason of anticancer chemotherapy [1,2]. This phenomenon is called multidrug resistance (MDR) and generally consists of acquired resistance as a result of chemotherapeutic treatment. MDR is mainly due to overexpression of some energy dependent transmembrane proteins belonging to the ABC (ATP Binding Cassette) transporter family [3,4]. These proteins act as extrusion pumps and are responsible of an accelerated efflux of chemotherapeutic agents out of the cancer cells. As a consequence, their cellular concentration decreases and the drug efficacy is reduced. Among the ABC proteins, three transporters, P-glycoprotein (P-gp or ABCB1), the multidrug-resistance-associated protein-1 (MRP1 or ABCC1) and the breast cancer resistance protein (BCRP or ABCG2) are mainly associated with cancer MDR being overexpressed in many cell lines [5].

P-glycoprotein is the most studied and characterized ABC transporter. It is overexpressed in many tumors and its elevated expression has been considered the cause of the reduced chemotherapeutic responses in various cancer types including both blood and solid cancers [6,7]. For this reason, P-gp has been considered a possible target to overcome cancer multidrug resistance [8] and, in the last years, many P-gp modulators have been designed and synthesized. These compounds can be coadministered with classical antitumor drugs that are substrates of P-gp, restoring their therapeutic efficacy in resistant tumors [9]. Many compounds have been identified for this purpose, nevertheless few of them have reached clinical trials [10]. The main obstacle is the widespread tissue distribution of P-gp and its physiological role in the regulation of permeability of biological membranes, in controlling the secretion of physiologically important lipophilic molecules and in the extrusion of xenobiotics that entered the organism [11,12]. Therefore, P-gp modulators could interfere with the pharmacokinetics of other substances, including coadministered chemotherapeutic agents [13]. For this reason, an ideal MDR reverser should be a P-gp modulator able to inhibit selectively the efflux of anticancer drugs in tumor cells without affecting the normal tissue membrane permeability.

Carbonic anhydrases (CAs, EC4.2.1.1) are metalloenzymes that catalyze the conversion of carbon dioxide to bicarbonate and a proton. They play a crucial role for the maintenance of pH homeostasis in the body and also have a metabolic function in several biosynthetic processes [14,15]. Therefore, CAs are involved in a multitude of physiological and pathological processes. Human CAs (hCAs) comprise of 15 different α-CA isoforms varying for their tissue distribution and cellular localization (membrane, cytosol and mitochondria) [14]. Among these isoforms, CA IX and XII are extracellular, membrane bound CAs associated with tumor progression and metastases formation [15].

The overexpression of these two isoenzymes is induced in most hypoxic solid tumors by the hypoxia inducible factor 1α (HIF-1α) [16,17,18]. The translocation of HIF-1α from the cytosol to the nucleus leads to the formation of an active transcription factor, which induces the overexpression of proteins involved in the proliferation and invasion of hypoxic tumor [19,20,21,22]. The overexpression of these proteins shifts the metabolism of tumor cells toward an anaerobic fermentation with the prevalence of glycolysis with respect to oxidative phosphorylation. The glycolytic pathway leads to the production of organic acids, which are extruded from the cells leading to an extracellular acidification. This condition is also supported by the extrusion of protons, produced by carbon dioxide hydration catalyzed by the transmembrane CA isoenzymes IX and XII, and the intracellular retention of bicarbonate ions. Thus, in cancer cells CA IX and XII are responsible for maintaining an alkaline intracellular pH and an extracellular acidosis that is beneficial for the growth of tumor cells but impairs the growth of normal cells [23,24]. It has recently been shown [25] that even in normoxia the HIF-1α factor accumulates in the cell cytoplasm and allows the transcription of some of its target genes including CA IX and CA XII, acting as a pH sensor.

Kopecka and colleagues reported in recent work that the activity of the efflux transmembrane transporter P-gp could be modulated by CA XII [26]. The P-gp efflux activity is known to be influenced by intracellular pH, as the optimal pH at which P-gp operates is slightly alkaline. This condition is maintained by the CA XII catalytic activity, which assures the suitable intra- and extracellular pH in tumor cells overexpressing it. CA XII is highly expressed in some chemoresistant P-gp positive cancer cells [26] and its activity is critical for the P-gp efflux function and contribution to MDR. In fact, there are recent evidences that the reduction of pH, caused by the inhibition of CA XII, produces a significant decrease in P-gp ATPase activity [26,27].

Compounds endowed with dual P-gp/CA XII inhibitory activity could thus be useful as chemosensitizers able to overcome P-gp mediated MDR in cancer cells. This synergistic mechanism should allow such compounds to be selective P-gp inhibitors for resistant tumor cells. Notably, they may have low interference with the physiological activity of P-gp in the regulation of the membrane permeability of normal tissues.

Acetazolamide and other classical CA inhibitors contain an aromatic sulfonamide moiety, which coordinates the zinc ion buried at the bottom of the conical CA active site [28]. The aromatic and heteroaromatic sulfonamides are non-selective inhibitors and, therefore, able to block both the transmembrane isoforms CA IX and CA XII, and the physiological relevant cytosolic isoenzymes CA I and CA II, thus leading to side effects [28]. Coumarin derivatives are a relatively new class of CA inhibitors [29,30,31] that show high selectivity for inhibiting the tumor associated CA IX and CA XII isoforms. This selectivity is explained by their particular binding mode; in fact, the hydrolyzed coumarin moiety is able to obstruct the active site cavity, binding at its entrance, which is the most variable part of the site among the various hCAs [32].

**Chart 1:**
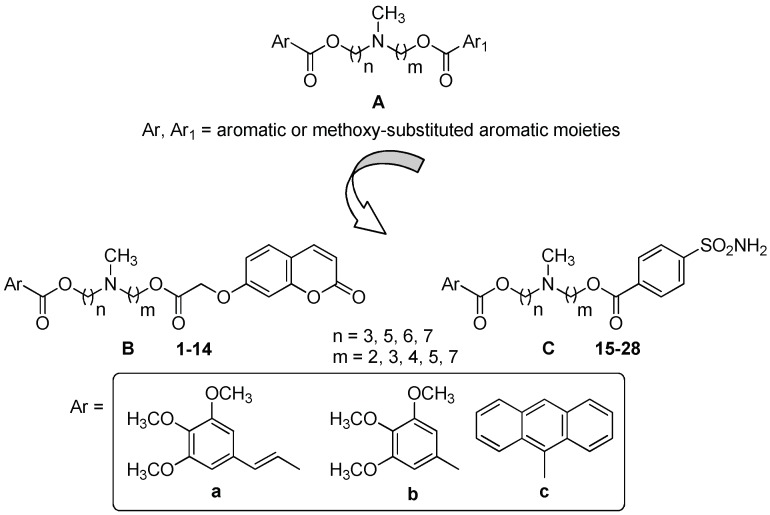
General structures of the leads and newly synthesized compounds.

The aim of this study was to obtain compounds able to overcome the P-gp mediated MDR in cancer cells by a synergistic mechanism of interference with CA XII and P-gp activity. For this aim, we designed and synthesized a new series of hybrid inhibitors characterized by the presence of both P-gp and CA XII binding moieties in order to possess a dual P-gp/CA XII inhibitory effect. We incorporated coumarin or benzene sulfonamide moieties in a typical scaffold of P-glycoprotein ligands that were previously studied by some of us. For many years one of our research group has been involved in the study of P-gp ligands with the aim to discover potent MDR modulators [33,34,35,36,37,38,39]. For this purpose, we designed and synthesized several compounds carrying the structural elements considered important for P-gp interaction, such as high lipophilicity, the presence of hydrogen bond acceptor groups, aromatic moieties and one or more protonable nitrogen atoms [40]. The structure of these molecules is characterized by the presence of a basic nitrogen atom linked to aromatic ester residues by spacers of different flexibility and length [33,34,35,36,37,38,39]. Most of these compounds are *N*,*N*-bis(alkanol)amine aryl diesters that were potent and efficient P-gp-dependent multidrug resistance reversers on the doxorubicin-resistant erythroleukemia K562 cell line (Structure A, Chart 1) [35,36,37,38,39].

In this study we report a new series of *N*,*N*-bis(alkanol)amine aryl diesters (**1**–**28**), incorporating two polymethylenic chains of variable length, carrying the (*E*)-3-(3,4,5-trimethoxyphenyl)vinyl, 3,4,5-trimethoxyphenyl or the anthracene residues (**a**, **b** and **c**), combined with coumarin or benzene sulfonamide moieties (Structures B and C, Chart 1).

The synthesized compounds were evaluated for their P-gp modulating activity by measuring the uptake increase of the specific P-gp substrate rhodamine-123 (Rhd 123) on doxorubicin-resistant erythroleukemia K562 cells (K562/DOX) that overexpress the membrane P-gp. The CA inhibition efficacy of compounds **1**–**28** was evaluated on the tumor associated hCA IX and hCA XII and on the physiological relevant cytosolic hCA I and II isoforms, to verify their CA isoforms selectivity profiles.

The expression levels of the two transmembrane proteins, P-gp and CA XII, were evaluated by real time polymerase chain reaction (RT-PCR) analysis, on resistant K562/DOX and parental K562 cell lines. The results showed a substantial increase in the mRNA expression level for the MDR1 gene but only a low increase in the mRNA expression level for the CA XII gene on the resistant K562/DOX line. Therefore, to prove the synergistic effect on the MDR reversal activity due to dual P-gp/CA XII inhibition, the selected compounds were evaluated by the Rhd 123 uptake test, on human colorectal carcinoma LoVo/DOX cells, which overexpress both P-gp and CA XII, as confirmed by the RT-PCR analysis performed on both resistant and parental cell lines (LoVo/DOX, LoVo). The P-gp expression level in both resistant K562/DOX and LoVo/DOX cells was also investigated using flow cytometric analysis with a P-gp-specific antibody.

Finally, the selected compounds were evaluated by the doxorubicin cytotoxicity enhancement test on K562/DOX and LoVo/DOX cells. Moreover, the susceptibility of derivatives **1**–**28** to spontaneous hydrolysis was assessed in phosphate buffer solution.

## 2. Results and Discussion

### 2.1. Chemistry

**Scheme 1:**
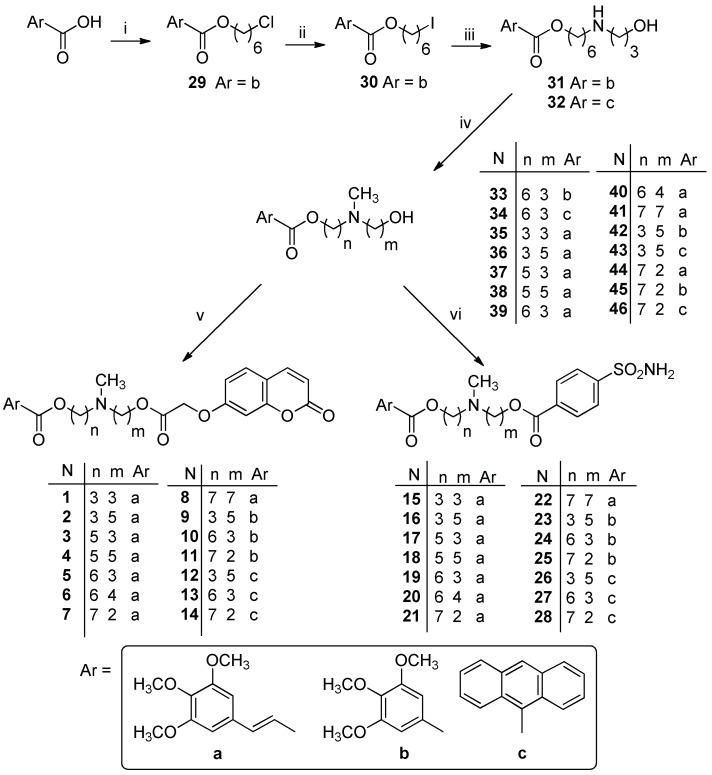
Reagents and conditions: (i) 6-chlorohexan-1-ol, EDC hydrochloride, DMAP and CH_2_Cl_2_; (ii) NaI, acetone; (iii) H_2_N(CH_2_)_3_OH and CH_3_CN; (iv) HCOOH/HCHO, EtOH; (v) 2-((2-Oxo-2*H*-chromen-7-yl)oxy)acetic acid (**48**), EDC hydrochloride, HOBt and CH_3_CN; (vi) 4-sulfamoylbenzoyl chloride, ethanol-free CHCl_3_.

**Scheme 2:**
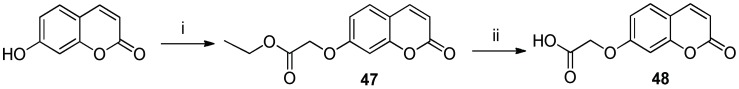
Reagents and conditions: (i) BrCH_2_COOC_2_H_5_, K_2_CO_3_, acetone and (ii) NaOH 10%.

Compounds **1**–**28** were prepared using a reaction pathway (Scheme 1) that has been previously applied to the synthesis of analogues with the general formula A (Chart 1) [35,36,37,38]. In this work, some new intermediates have been synthesized for preparing all the newly designed hybrids. Thus, chloroester **29** was obtained by esterification of 6-chlorohexan-1-ol with the commercially available 3,4,5-trimethoxybenzoic acid using EDC hydrochloride (*N*-(3-dimethylaminopropyl)-*N*′-ethylcarbodiimide hydrochloride) and DMAP (4-dimethylaminopyridine) in anhydrous CH_2_Cl_2_. The chloroalkyl ester was transformed in the corresponding iodo derivative **30** using NaI in acetone, in order to achieve higher yields in the following reactions. Treatment of **30** with 3-aminopropan-1-ol in acetonitrile gave the desired secondary amine **31**. The same reaction performed on the already described 6-iodohexyl anthracene-9-carboxylate [38], gave **32**. These compounds were alkylated by reductive methylation with HCOOH/HCHO to give the corresponding tertiary amines **33** and **34**. Final compounds **1–14** were obtained by esterification of **33, 34** and the previously reported analogues **35**–**46** [35,36,37,38] with 2-((2-oxo-2*H*-chromen-7-yl)oxy)acetic acid (**48**) (described in Scheme 2) using EDC hydrochloride and HOBt (1-hydroxybenzotriazole hydrate) in anhydrous CH_3_CN. In turn, compounds **15**–**28** were prepared by esterification of **33–46** with 4-sulfamoylbenzoyl chloride obtained from 4-sulfamoylbenzoic acid by reaction with SOCl_2_.

2-((2-Oxo-2*H*-chromen-7-yl)oxy)acetic acid (**48**) was synthesized as reported in Scheme 2. Alkylation of commercially available 7-hydroxy-2*H*-chromen-2-one with ethyl bromoacetate gave ester **47**, which was hydrolyzed under alkaline conditions to obtain **48**.

### 2.2. Chemical Stability

In order to evaluate the chemical stability of our compounds during biological tests, a study on the susceptibility to spontaneous hydrolysis of derivatives **1**–**28** was planned. For this purpose, a series of experiments were carried out adding a known amount of analyte in phosphate buffer solution (PBS). The samples were analyzed by LC–MS/MS method operating in the multiple reaction monitoring (MRM) mode [39]. The solution stability of each studied compound was verified by monitoring the variation of the analyte concentration at different incubation times. The processed raw data and the used LC–MS/MS methods are reported in the Supplementary Material.

The obtained results demonstrated that compounds **1**–**28** were stable in PBS showing a negligible decay rate (k values close to 0). Therefore, extremely high half-life values can be calculated for these derivatives. Since 240 min is the maximum time of observation in our experimental protocol, it is possible to assume that the half-lives of these hybrids should be greater than 240 min.

### 2.3. Rhodamine-123 Uptake Test on K562/DOX Cells

The ability of compounds **1**–**28** to inhibit the P-gp transport activity was evaluated by measuring the uptake of the specific P-gp substrate rhodamine-123 (Rhd 123) in K562/DOX cells, as described in Material and Methods.

K562 is a highly undifferentiated erythroleukemia cell line originally derived from a patient with chronic myelogenous leukemia [41]. The doxorubicin resistant K562/DOX cell line overexpresses almost exclusively the transporter membrane protein P-gp [42].

The P-gp inhibitory activity of compounds **1–28**, tested at 3 and 30 µM concentrations, are reported in Table 1 together with those of verapamil, used as standard inhibitor.

The new compounds were preliminary studied to evaluate their intrinsic cytotoxicity at 3 and 30 µM concentrations by the MTT (3-(4,5-dimethylthiazolyl-2)-2,5-diphenyltetrazolium bromide) assay [43] on the K562/DOX cells line. At the concentrations tested, all compounds showed a negligible intrinsic toxicity (about 20%, data not shown).

The results were expressed as the FR (fluorescence ratio), which is the ratio between the average fluorescence intensity of rhodamine in the presence and in the absence of modulators. Value 1 was attributed to the average fluorescence intensity of the samples exposed only to rhodamine.

The data reported in Table 1 indicate that at 3 µM concentration most compounds were inactive in the inhibition of the P-gp transport activity (FR values equal to or close to the unity), but **4**, **7**, **8**, **22** and **26** were able to increase the intracellular rhodamine uptake by at least twice o even more (FR = 1.99, 2.08, 2.33, 2.85 and 2.10, respectively), and compound **5** was active as verapamil (FR = 3.67 and 3.30, respectively).

At 30 µM concentration a significant increase in the intracellular rhodamine fluorescence intensity was observed for all compounds and many of them showed FR values in the 4.15–5.85 range. Notably, compounds **2**, **4**, **5** and **18** showed FR values higher than that of verapamil, tested at the same concentration (FR = 5.81, 5.45, 5.85 and 4.99, respectively, vs. FR = 4.71 of verapamil). On the contrary, the standard CA inhibitor acetazolamide did not show any effect on K562/DOX cells at both the concentrations used.

The results showed in Table 1 indicate that the introduction of a coumarin or a benzene sulfonamide group maintains effective P-glycoprotein inhibitory ability, even if the potency is lower compared to the previously synthesized analogues carrying on both sides the aromatic moieties **a**–**c** reported in Chart 1 (General structure A) [38].

Notably, compounds from the coumarin series (Structure B, Table 1) were in general more potent than those from the benzene sulfonamide series (Structure C, Table 1; compare **2** vs. **16**, **4** vs. **18** and **5** vs. **19**). On the other hand, considering the nature of the Ar group, compounds bearing a (*E*)-3-(3,4,5-trimethoxyphenyl)vinyl moiety were in general more active. Disappointingly, and differently from what found in previous series [38], a clear influence of the length of the linkers on the activity was not observed with the new derivatives reported here. 

### 2.4. CA Inhibitory Activity

The CA inhibitory efficacy of compounds **1**–**28** was evaluated on four human CA isoforms, the two cytosolic hCA I and II and the transmembrane tumor-associate hCA IX and XII isoforms. The hCA inhibition data of the new compounds were determined by a Stopped-Flow CO_2_ hydrase assay [44] and are reported in Table 1 together with those of the sulfonamide acetazolamide (AAZ), used as a standard inhibitor.

Derivatives **1**–**14**, characterized by the presence of a coumarin scaffold, inhibited both hCA IX and XII isoforms at nanomolar concentrations, while they were inactive against the off-target isoforms hCA I and II, in accord with the selectivity usually displayed by CA inhibitors decorated with this aromatic moiety. In addition, this series of compounds showed lower Ki values on hCA XII (Ki = 6.4–48.8 nM) than on hCA IX (Ki = 24.2–150.8 nM). Some compounds (**1**, **9**, **10** and **11**) displayed an interesting 12–15 fold preference for hCA XII with respect to hCA IX: compounds **9**, **10** and **11** are 3,4,5-trimethoxyphenyl derivatives while **1** carries a *(E)-*3-(3,4,5-trimethoxyphenyl)vinyl moiety. The latter group can also confer high potency, since **5** (Ki 6.4 nM) was the most potent compound on hCA XII of this series. On the contrary, derivatives with an anthracene moiety were, in general, less potent and selective, with the exception of **13**, which was nine times more active on hCA XII than on hCA IX. The linkers’ length seemed not to be crucial for the interaction with hCA XII: for instance, compounds **1** having the shortest linkers (n, m = 3) were equipotent with **8,** which showed the longest linkers on both arms (n, m = 7). On hCA IX, if we take into account the compounds having the same Ar group (**1**–**8**, Ar = **a**) only the short 3 + 3 linker seemed detrimental for activity, since compound **1** was 3–6 times less potent than **2**–**7**.

As expected, compounds **15**–**28**, incorporating a benzene sulfonamide moiety, were able to inhibit all four isoforms, even if with different selectivity profiles, showing Ki values in the range of 20.6–241.2 nM and 8.0–96.7 nM ranges for hCA IX and XII isoforms, respectively. Almost all compounds were more effective CA XII than CA IX inhibitors, with the exception of **23** and **24** (twice more potent on hCA IX than on hCA XII), and of **20**, **27** and **28**, which showed similar K_i_ values on both isoforms.

The selectivity profiles towards hCA XII with respect to the two hCA I and II isoforms were quite different. Compounds **16**, **19**, **21**, **23**, **26**, **27** and **28** showed a preference for hCA XII vs. hCA I (4–20 times). In particular, compounds bearing an anthracene moiety (**26**, **27** and **28**) were the most selective ones.

Compounds **19**, **20** and **24** were the most potent on hCA II, with Ki = 8.4, 14.0 and 8.9 nM, respectively with an hCA II vs. hCA XII preference of 7, 3 and 6.5 times, respectively. Conversely, some hCA XII vs. hCA II selectivity (2–20 times) was displayed by compounds **16**, **17**, **18**, **22**, **23**, **25**, **26**, **27** and **28**. Also in this case compounds bearing an anthracene moiety (**26**, **27** and **28**) were all quite selective. 

In conclusion, the data reported in Table 1 show that many compounds acted as effective CA inhibitors, although the Ar moiety (**a**-**c**) or the linkers’ length did not clearly influence their activity on the four hCA isoforms. Notably, several coumarin derivatives (**1**, **2**, **5**, **7**–**11** and **13**) and one sulfonamide derivative (**25**) had Ki values on hCA XII in the low nanomolar range. On the contrary, sulfonamides **19**, **20** and **24** displayed potent, although undesired, inhibitory activity on hCA II (Ki values between 8.4 and 14 nM).

### 2.5. P-Glycoprotein and CA XII Expression in K562/DOX and LoVo/DOX Cells

The gene expression levels of P-gp and CA XII were checked by real-time PCR analysis in K562 leukemic and LoVo colon carcinoma cells, both in sensitive and resistant lines. This test was based on the quantitative assessment of the presence of transcripts, i.e., messenger RNA molecules encoding the two proteins [45]. The real-time PCR analysis indicated a substantial increase in the mRNA expression level for the MDR1 gene on the resistant leukemic line, K562/DOX, with respect to the parental counterpart (about 173 times). On the contrary, a low increase in the mRNA expression level for the CA XII gene was observed on the resistant K562/DOX line compared with the parental one (about two times). The doxorubicin resistant colon carcinoma cell line LoVo/DOX, showed an increase in the mRNA expression level 53 times higher than the parental counterpart for the MDR1 gene. In this cell line an increase in the mRNA expression level for the CA XII gene of 11 times was also observed. Details are reported in Material and Methods and Supplementary Material.

The P-glycoprotein expression level in resistant K562/DOX and LoVo/DOX cells was also checked using flow cytometric analysis with the P-gp-specific monoclonal antibody Mab FITC conjugate CD243. The obtained results, reported as a fluorescence ratio between the resistant line and the parental counterpart, showed an increase in the protein expression of about 17.3 times in the K562/DOX line, and of about 8.6 times in the LoVo/DOX line. Details are reported in Material and Methods and Supplementary Material.

### 2.6. Rhodamine-123 Uptake Test on LoVo/DOX Cells

Based on the results on hCA inhibitory activity and on K562/DOX Rhd 123 uptake tests, compounds with the best profile in term of potency on P-gp and selectivity towards the hCA XII isoform were selected (coumarin **2**, **5**, **7** and **8**, and benzene sulfonamide **21**, **25** and **26** derivatives) for further evaluation on human colorectal carcinoma LoVo/DOX cells.

LoVo is a human colon adenocarcinoma cell line isolated from a metastatic nodule and its MDR variant, LoVo/DOX, was obtained from LoVo (parental line) by exposure to increasing concentrations of doxorubicin.

The selected compounds were preliminary studied at 3, 10 and 30 µM concentrations by the MTT (3-(4,5-dimethylthiazolyl-2)-2,5-diphenyltetrazolium bromide) assay [43] to evaluate their intrinsic cytotoxicity on LoVo/DOX cell line. The compounds displayed at 3 and 10 µM concentrations a negligible intrinsic toxicity (about 20%), that increased at the higher dose (22%–42%). Therefore, the 30 µM concentration was not used in the further studies and the rhodamine-123 uptake test on the colon carcinoma model (LoVo/DOX cells) was performed at 3 and 10 µM.

**Figure 1:**
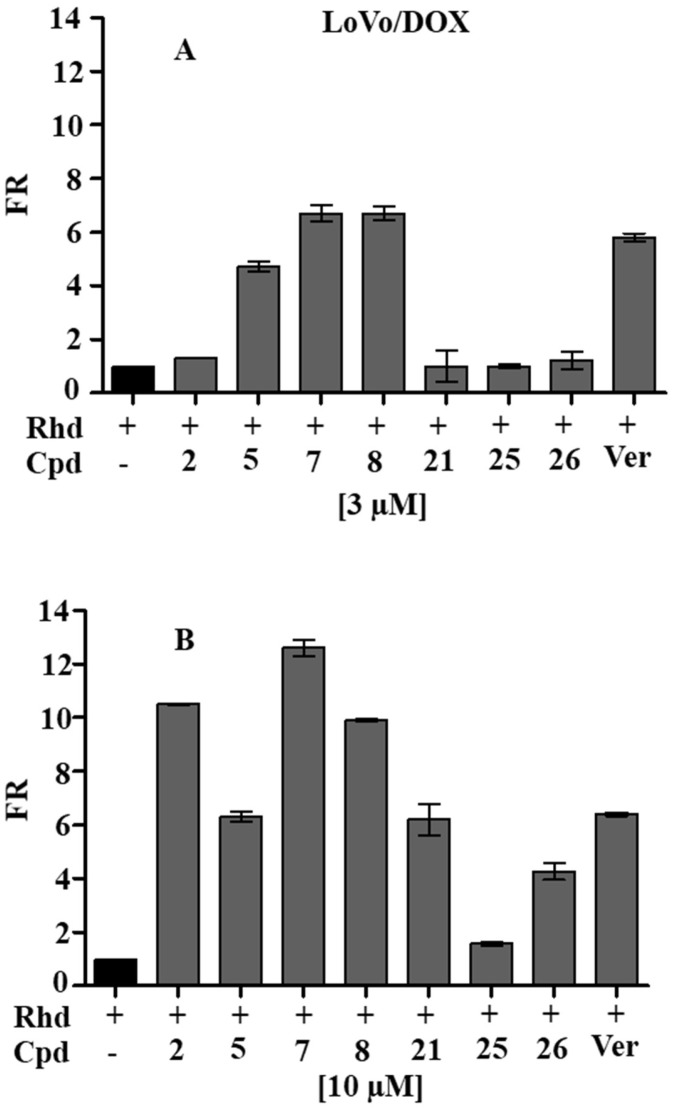
Fluorescence ratio (FR) values in LoVo/DOX cells incubated with rhodamine in the presence and in absence of modulators **2**, **5**, **7**, **8**, **21**, **25** and **26** and verapamil at 3 µM (panel **A**) and 10 µM (panel **B**) concentrations. Value 1 was attributed to the average fluorescence intensity of the samples exposed only to rhodamine.

The results, expressed as FR values, were reported as the histograms shown in Figure 1. At 3 µM, the intracellular fluorescence intensity of rhodamine varied in the 1.32–6.70 range for compounds **2**, **5**, **7** and **8**, but was negligible for **21**, **25** and **26** (FR values equal to or close to the unity; Figure 1, panel A). At 10 µM, instead, all molecules showed a significant increase in the intracellular rhodamine uptake. Especially, compounds **2**, **5**, **7** and **8** showed FR values of 10.50, 6.30, 12.60 and 9.90, respectively, that resulted equal to or higher than that of verapamil (6.40) tested at the same concentration (Figure 1, panel B).

These results confirmed the already observed behavior on K562/DOX cell line; in fact, also on LoVo/DOX cells, the coumarin derivatives were in general more potent in P-gp transport inhibition than compounds carrying the benzene sulfonamide moiety.

### 2.7. Enhancement of Doxorubicin Cytotoxicity

Compounds **2**, **5**, **7**, **8**, **21**, **25** and **26** were further evaluated, at 3 and 10 µM concentrations, to assess their effects on the doxorubicin cytotoxicity enhancement on K562/DOX and LoVo/DOX cell lines.

Doxorubicin is a P-gp substrate and is usually inactive in tumors overexpressing the pump. The extent of resistance to doxorubicin of K562/DOX and LoVo/DOX cells was established and expressed by the resistance index, as reported in Material and Methods.

In this test, doxorubicin was used at the low toxic concentrations that caused a 20% cell growth inhibition (IC_20_) on the two resistant lines (IC_20_ = 0.5 µM for K562/DOX cells and 0.3 µM for LoVo/DOX cells). 

On K562/DOX cells, all compounds at 3 µM had low or no effect in the enhancement of doxorubicin IC_20_ cytotoxicity, except compound **5,** which reduced the cell growth from 80% to 51.7%. At 10 µM, all tested compounds increased doxorubicin cytotoxicity with different extent. Coumarin derivatives **5**, **7** and **8** reduced the cell growth below 50%, in particular compound **5** reduced it to 25.1% (Table 2).

On LoVo/DOX cells, compounds **5**, **7** and **8** at 3 µM reduced the cell growth to 58.0, 61.0 and 68.0%, respectively, and the other derivatives had no effect.

At the higher concentration (10 µM) almost all compounds were able to increase the doxorubicin cytotoxicity. Notably, compounds **2**, **5**, **7** and **8** caused a reduction of the cells’ growth from 80% to 41.5%, 51.0%, 34.9% and 32.2%, respectively. The date reported in Table 2 indicate that, in general, the ability of these compounds on the doxorubicin cytotoxicity enhancement was higher on LoVo/DOX cells than on K562/DOX cells.

## 3. Material and Methods

### 3.1. Chemistry

All melting points were taken on a Büchi apparatus and were uncorrected. NMR spectra were recorded on a Bruker Avance 400 spectrometer (400 MHz for ^1^H NMR, 100 MHz for ^13^C NMR) using residual solvent such as chloroform (δ = 7.26) as the internal standard. ^1^H and ^13^C chemical shifts are expressed in ppm (δ) referring to TMS. Spectral data are reported using the following abbreviations: s = singlet, bs = broad singlet, d = doublet, dd = doublet of doublets, t = triplet and m = multiplet and coupling constants are reported in Hz, followed by integration. Chromatographic separations were performed on a silica gel column by flash chromatography (Kieselgel 40, 0.040–0.063 mm; Merck). Yields were given after purification, unless otherwise stated.

The high-resolution mass spectrometry (HRMS) analysis was performed with a Thermo Finnigan LTQ Orbitrap mass spectrometer equipped with an electrospray ionization source (ESI). The accurate mass measure was carried out by introducing, via syringe pump at 10 μL min^-1^, the sample solution (1.0 μg mL^-1^ in mQ water: acetonitrile 50:50), and the signal of the positive ions was acquired. The proposed experimental conditions allowed to monitoring the protonated molecules of studied compounds ([M + H]^+^ species), that they were measured with a proper dwell time to achieve 60,000 units of resolution at full width at half maximum (FWHM). The elemental composition of compounds was calculated on the basis of their measured accurate masses, accepting only results with an attribution error less than 2.5 ppm and a not integer RDB (double bond/ring equivalents) value, in order to consider only the protonated species [46].

Compounds were named following IUPAC rules as applied by ChemBioDraw Ultra 14.0 software (Cambridgesoft, Perkin Elmer, Milan, Italy). When reactions were performed in anhydrous conditions, the mixtures were maintained under nitrogen. Free bases **15–28** were transformed into the hydrochloride by treatment with a solution of acetyl chloride (1.1 equivalents) in anhydrous CH_3_OH. The salts were crystallized from abs. ethanol/petroleum ether.

*6-Chlorohexyl 3,4,5-trimethoxybenzoate***29**. A solution of 909 mg (4.28 mmol) of 3,4,5-trimethoxybenzoic acid in 25 mL of anhydrous CH_2_Cl_2_ was cooled at 0 °C and 0.33 mL (2.86 mmol) of 6-chlorohexan-1-ol, 0.28 g (2.28 mmol) of DMAP and 0.98 g (5.14 mmol) of EDC hydrochloride were added in this order. The reaction mixture was stirred at 0 °C for 1 h and at room temperature for 48 h, then treated with CH_2_Cl_2_. The organic layer was washed three times with water and twice with a saturated solution of NaHCO_3_. After drying with Na_2_SO_4_, the solvent was removed under reduced pressure. The crude product was then purified by flash chromatography using CH_2_Cl_2_/CH_3_OH 98:2 as eluent, yielding 943 mg (100%) of the desired compound as an oil. ^1^H-NMR (400 MHz, CDCl_3_) δ: 7.22 (s, 2H, CH); 4.24 (t, *J* = 6.8 Hz, 2H, OCH_2_); 3.84 (s, 6H, OCH_3_); 3.83 (s, 3H, OCH_3_); 3.47 (t, *J* = 6.4 Hz, 2H, CH_2_Cl); 1.75–1.69 (m, 4H, CH_2_); 1.49–1.39 (m, 4H, CH_2_) ppm.

*6-Iodohexyl 3,4,5-trimethoxybenzoate***30**. To a solution of 1.069 g (3.24 mmol) of compound **29** in 15 mL of acetone, NaI (2.90 g, 19.4 mmol) was added, and the resulting mixture was maintained at reflux in the dark for 19 h. The reaction mixture was cooled to room temperature, and the solvent was removed under reduced pressure; the residue was dissolved in CH_2_Cl_2_ and washed with water. The organic layer was dried with Na_2_SO_4_, and the solvent was removed under reduced pressure yielding 1.27 g (93%) of the desired compound **30** as an oil, which was used as such for the next reaction. ^1^H-NMR (400 MHz, CDCl_3_) δ: 7.21 (s, 2H, CH); 4.24 (t, *J* = 6.4 Hz, 2H, OCH_2_); 3.83 (s, 6H, OCH_3_); 3.82 (s, 3H, OCH_3_); 3.11 (t, *J* = 6.8 Hz, 2H, CH_2_I); 1.77–1.69 (m, 4H, CH_2_); 1.40–1.35 (m, 4H, CH_2_) ppm. 

*6-((3-Hydroxypropyl)amino)hexyl 3,4,5-trimethoxybenzoate***31**. To a solution of 1.27 g (3.00 mmol) of compound **30** in 20 mL of anhydrous CH_3_CN, 0.42 g (3.00 mmol) of K_2_CO_3_ and 0.70 mL (9.00 mmol) of 3-aminopropan-1-ol were added. The mixture was heated at 80 °C for 18 h. Then the solvent was removed under reduced pressure; the residue was treated with CH_2_Cl_2_ and the organic layer was washed with 10% NaOH solution. After drying with Na_2_SO_4_, the solvent was removed under reduced pressure and the residue purified by flash chromatography using CH_2_Cl_2_/CH_3_OH/NH_4_OH 90:10:1 as eluent. The desired compound **31** (0.950 g, 86%) was obtained as an oil. ^1^H-NMR (400 MHz, CDCl_3_) δ: 7.22 (s, 2H, CH); 4.24 (t, *J* = 6.8 Hz, 2H, OCH_2_); 3.85 (s, 6H, OCH_3_); 3.83 (s, 3H, OCH_3_); 3.73 (t, *J* = 5.2 Hz, 2H, CH_2_OH); 3.45 (bs, 2H, NH and OH); 2.82 (t, *J* = 5.6 Hz, 2H, NCH_2_); 2.56 (t, *J* = 7.2 Hz, 2H, NCH_2_); 1.72–1.63 (m, 4H, CH_2_); 1.49–1.33 (m, 6H, CH_2_) ppm.

*6-((3-Hydroxypropyl)amino)hexyl anthracene-9-carboxylate***32**. Following the same procedure described for compound **31**, starting from 1.44 g (3.34 mmol) of 6-iodohexyl anthracene-9-carboxylate [38], compound **32** (0.766 g, 60%) was obtained as an oil. ^1^H-NMR (400 MHz, CDCl_3_) δ: 8.44 (s, 1H, CH); 7.98–7.93 (m, 4H, CH); 7.50–7.40 (m, 4H, CH); 4.55 (t, *J* = 6.8 Hz, 2H, OCH_2_); 3.67 (t, *J* = 5.2 Hz, 2H, CH_2_OH); 2.70 (t, *J* = 5.6 Hz, 2H, NCH_2_); 2.49 (t, *J* = 6.8 Hz, 2H, NCH_2_); 1.83–1.77 (m, 2H, CH_2_); 1.63–1.57 (m, 2H, CH_2_); 1.42–1.31 (m, 6H, CH_2_) ppm.

*6-((3-Hydroxypropyl)(methyl)amino)hexyl 3,4,5-trimethoxybenzoate***33**. Compound **31** (0.238 g, 0.65 mmol) was dissolved in 5 mL of anhydrous ethanol, then HCOOH (0.40 mL) and 37% HCHO solution (0.09 mL, 3.23 mmol) were added. The mixture was heated at 80 °C for 4–5 h and concentrated in vacuo. The residue was then dissolved in CH_2_Cl_2_ and the organic layer was washed with 10% NaOH solution. After drying with Na_2_SO_4_, the solvent was removed under reduced pressure, and the residue purified by flash chromatography using CH_2_Cl_2_/CH_3_OH/NH_4_OH 90:10:1 as eluent. The desired compound **33** (0.169 g, 68%) was obtained as an oil. ^1^H-NMR (400 MHz, CDCl_3_) δ: 7.24 (s, 2H, CH); 4.25 (t, *J* = 6.8 Hz, 2H, OCH_2_); 3.86 (s, 6H, OCH_3_); 3.85 (s, 3H, OCH_3_); 3.74 (t, *J* = 5.2 Hz, 2H, CH_2_OH); 2.55 (t, *J* = 5.6 Hz, 2H, NCH_2_); 2.33 (t, *J* = 7.2 Hz, 2H, NCH_2_); 2.20 (s, 3H, NCH_3_); 1.74–1.63 (m, 4H, CH_2_); 1.51–1.30 (m, 6H, CH_2_) ppm.

*6-((3-Hydroxypropyl)(methyl)amino)hexyl anthracene-9-carboxylate***34.** Following the same procedure described for compound **33**, starting from 0.224 (0.59 mmol) of **32**, compound **34** (0.226 g, 98%) was obtained as an oil. ^1^H-NMR (400 MHz, CDCl_3_) δ: 8.39 (s, 1H, CH); 8.01 (d, *J* =8.8 Hz, 2H, CH); 7.90 (d, *J* = 8.8 Hz, 2H, CH); 7.50–7.38 (m, 4H, CH); 4.56 (t, *J* = 6.8 Hz, 2H, OCH_2_); 3.71 (t, *J* = 5.2 Hz, 2H, CH_2_OH); 2.43 (t, *J* = 5.6 Hz, 2H, NCH_2_); 2.23 (t, *J* = 7.2 Hz, 2H, NCH_2_); 2.12 (s, 3H, NCH_3_); 1.85–1.78 (m, 2H, CH_2_); 1.61–1.50 (m, 2H, CH_2_); 1.49–1.28 (m, 6H, CH_2_) ppm.

*Ethyl 2-((2-oxo-2H-chromen-7-yl)oxy)acetate***47**. To a solution of 7-hydroxy-2*H*-chromen-2-one (240 mg, 0.97 mmol) in 8 mL of acetone, K_2_CO_3_ (228 mg, 1.63 mmol) was added. The mixture was stirred at room temperature for 15 min, and then ethyl bromoacetate (0.18 mL, 1.63 mmol) dissolved in 6 mL acetone was added dropwise. The reaction mixture was heated at 58 °C for 6 h and, after cooling, water (10 mL) was added. The suspension was filtered under vacuum and the solid obtained was dried under vacuum. The title compound was obtained as a white solid. Yield: 89%, mp 107–110 °C. ^1^H-NMR (400 MHz, CDCl_3_) δ: 7.64 (d, *J* = 9.6 Hz, 1H, CH); 7.40 (d, *J* = 8.4 Hz, 1H, CH); 6.89 (dd, *J* = 8.4, 2.2 Hz, 1H, CH); 6.78 (d, *J* = 2.2 Hz, 1H, CH); 6.28 (d, *J* = 9.6 Hz, 1H, CH); 4.68 (s, 2H, OCH_2_); 4.30 (q, *J* = 7.2 Hz, 2H, OCH_2_); 1.32 (t, *J* = 7.2 Hz, 3H, CH_3_) ppm.

*2-((2-Oxo-2H-chromen-7-yl)oxy)acetic acid***48**. Ten milliliters of 10% NaOH solution were added to 225 mg (0.95 mmol) of **47**. The mixture was heated at 100 °C for 3 h. After cooling, the solution was acidified with HCl 2N and the solid was filtered under vacuum and then dried. The title compound was obtained as a white solid. Yield: 100%, mp 220–223 °C. ^1^H-NMR (400 MHz, DMSO-d_6_) δ: 7.98 (d, *J* = 9.6, Hz 1H, CH); 7.62 (d, *J* = 8.4 Hz, 1H, CH); 6.94 (s, 1H, CH); 6.93 (d, *J* = 8.4 Hz, 1H, CH); 6.28 (d, *J* = 9.6 Hz, 1H, CH); 4.81 (s, 2H, OCH_2_) ppm.

#### 3.1.1. General Procedure for the Synthesis of Diester Compounds **1**–**14**

To a solution of **48** (0.26 mmol) in 25 mL of anhydrous CH_3_CN, 0.33 mmol of EDC hydrochloride and 0.33 mmol of HOBt were added. The mixture was stirred at room temperature for 1 h, and then the suitable (hydroxyalkyl) methylaminoester **33–46** (0.22 mmol) dissolved in 5 mL of anhydrous CH_3_CN was added. The reaction mixture was stirred for 4 h at room temperature and the solvent was removed under reduce pressure. Then CH_2_Cl_2_ was added and the organic layer was washed twice with a saturated solution of NaHCO_3_. After drying with Na_2_SO_4_, the solvent was removed under reduced pressure. The crude product was then purified by flash chromatography, using the proper eluting system, yielding the desired compound as an oil.

*(E)-3-(methyl(3-(2-((2-oxo-2H-chromen-7-yl)oxy)acetoxy)propyl)amino)propyl 3-(3,4,5-trimethoxyphenyl)acrylate***1**. From (hydroxyalkyl)methylaminoester **35 [35]**. Oil. Chromatographic eluent: CH_2_Cl_2_/CH_3_OH 95:5. Yield: 37%. ^1^H-NMR (400 MHz, CDCl_3_) δ: 7.63 (d, *J* = 9.4 Hz, 1H, CH); 7.58 (d, *J* = 16.0 Hz, 1H, C*H*=CH); 7.38 (d, *J* = 8.4 Hz, 1H, CH); 6.87 (dd, *J* = 8.4, 2.2 Hz, 1H, CH); 6.77 (d, *J* = 2.2 Hz, 1H, CH); 6.74 (s, 2H, CH); 6.33 (d, *J* = 16.0 Hz, 1H, C*H*=CH); 6.26 (d, *J* = 9.4 Hz, 1H, CH); 4.68 (s, 2H, OCH_2_); 4.30–4.22 (m, 4H, OCH_2_); 3.88 (s, 6H, OCH_3_); 3.87 (s, 3H, OCH_3_); 2.47–2.35 (m, 4H, NCH_2_); 2.22 (s, 3H, NCH_3_); 1.87–1.81 (m, 4H, CH_2_) ppm. ^13^C-NMR (100 MHz, CDCl_3_) δ: 167.97 (C); 166.95 (C); 160.84 (C); 155.62 (C); 153.44 (C); 144.73 (CH); 143.16 (CH); 129.88 (C); 128.97 (CH); 117.31 (CH); 113.78 (CH); 133.33 (C); 112.80 (CH); 105.27 (CH); 101.70 (CH); 65.34 (CH_2_); 64.02 (CH_2_); 62.85 (CH_2_); 60.95 (OCH_3_); 56.17 (OCH_3_); 54.20 (CH_2_); 53.85 (CH_2_)_;_ 41.95 (NCH_3_); 26.67 (CH_2_); 26.47 (CH_2_) ppm. ESI-HRMS (*m*/*z*) calculated for [M + H]^+^ ion species C_30_H_36_NO_10_ = 570.2334, found 570.2340.

*(E)-3-(methyl(5-(2-((2-oxo-2H-chromen-7-yl)oxy)acetoxy)pentyl)amino)propyl 3-(3,4,5-trimethoxyphenyl)acrylate***2**. From **36** [37]. Oil. Chromatographic eluent: CH_2_Cl_2_/CH_3_OH 93:7. Yield: 29%. ^1^H-NMR (400 MHz, CDCl_3_) δ: 7.60 (d, *J* = 9.6 Hz, 1H, CH); 7.56 (d, *J* = 16.0 Hz, 1H, C*H*=CH); 7.36 (d, *J* = 8.4 Hz, 1H, CH); 6.85 (dd, *J* = 8.4, 2.2 Hz, 1H, CH); 6.74 (d, *J* = 2.2 Hz, 1H, CH); 6.72 (s, 2H, CH); 6.31 (d, *J* = 16.0 Hz, 1H, C*H*=CH); 6.23 (d, *J* = 9.6 Hz, 1H, CH); 4.66 (s, 2H, OCH_2_); 4.23–4.16 (m, 4H, OCH_2_); 3.85 (s, 6H, OCH_3_); 3.84 (s, 3H, OCH_3_); 2.42 (t, *J* = 6.8 Hz, 2H, NCH_2_); 2.30 (t, *J* = 6.8 Hz, 2H, NCH_2_); 2.19 (s, 3H, NCH_3_); 1.88–1.83 (m, 2H, CH_2_); 1.71–1.60 (m, 2H, CH_2_); 1.50–1.40 (m, 2H, CH_2_); 1.38–1.28 (m, 2H, CH_2_) ppm. ^13^C-NMR (100 MHz, CDCl_3_) δ: 167.98 (C); 166.92 (C); 160.81 (C); 155.63 (C); 153.41 (C); 144.65 (CH); 143.18 (CH); 129.87 (C); 128.95 (CH); 117.33 (CH); 113.70 (CH); 113.29 (C); 112.79 (CH); 105.26 (CH); 101.71 (CH); 65.63 (CH_2_); 65.32 (CH_2_); 62.98 (CH_2_); 60.92 (OCH_3_); 57.45 (CH_2_); 56.15 (OCH_3_); 54.19 (CH_2_); 42.08 (NCH_3_); 28.42 (CH_2_); 26.87 (CH_2_); 26.67 (CH_2_); 23.67 (CH_2_) ppm. ESI-HRMS (*m*/*z*) calculated for [M + H]^+^ ion species C_32_H_40_NO_10_ = 598.2647, found 598.2651.

*(E)-5-(methyl(3-(2-((2-oxo-2H-chromen-7-yl)oxy)acetoxy)propyl)amino)pentyl 3-(3,4,5-trimethoxyphenyl)acrylate***3**. From **37 [37]**. Oil. Chromatographic eluent: CH_2_Cl_2_/CH_3_OH 95:5. Yield: 33%. ^1^H-NMR (400 MHz, CDCl_3_) δ: 7.59 (d, *J* = 9.6 Hz, 1H, CH); 7.53 (d, *J* = 15.6 Hz, 1H, CH=CH); 7.34 (d, *J* = 8.4 Hz, 1H, CH); 6.82 (dd, *J* = 8.4, 2.2 Hz, 1H, CH); 6.72 (d, *J* = 2.2 Hz, 1H, CH); 6.70 (s, 2H, CH); 6.30 (d, *J* = 15.6 Hz, 1H, CH=CH); 6.21 (d, *J* = 9.6 Hz, 1H, CH); 4.64 (s, 2H, OCH_2_); 4.22 (t, *J* = 6.4 Hz, 2H, OCH_2_); 4.15 (t, *J* = 6.4 Hz, 2H, OCH_2_); 3.83 (s, 6H, OCH_3_); 3.82 (s, 3H, OCH_3_); 2.34 (t, *J* = 6.8 Hz, 2H, NCH_2_); 2.29 (t, *J* = 7.2 Hz, 2H, NCH_2_); 2.15 (s, 3H, NCH_3_); 1.82–1.75 (m, 2H, CH_2_); 1.69–1.64 (m, 2H, CH_2_); 1.52–1.32 (m, 4H, CH_2_) ppm. ^13^C-NMR (100 MHz, CDCl_3_) δ: 167.95 (C); 166.97 (C); 160.77 (C); 155.61 (C); 153.38 (C); 144.58 (CH); 143.20 (CH); 140.04 (C); 129.90 (C); 128.98 (CH); 117.41 (CH); 113.68 (CH); 113.29 (C); 112.73 (CH); 105.21 (CH); 101.68 (CH); 65.29 (CH_2_); 64.47 (CH_2_); 64.06 (CH_2_); 60.90 (OCH_3_); 57.51 (CH_2_); 56.12 (OCH_3_); 53.88 (CH_2_); 41.97 (NCH_3_); 28.64 (CH_2_); 26.81 (CH_2_); 26.37 (CH_2_); 23.81 (CH_2_) ppm. ESI-HRMS (*m*/*z*) calculated for [M + H]^+^ ion species C_32_H_40_NO_10_ = 598.2647, found 598.2642.

*(E)-5-(methyl(5-(2-((2-oxo-2H-chromen-7-yl)oxy)acetoxy)pentyl)amino)pentyl 3-(3,4,5-trimethoxyphenyl)acrylate***4**. From **38 [35]**. Oil. Chromatographic eluent: CH_2_Cl_2_/CH_3_OH 95:5. Yield: 46%. ^1^H-NMR (400 MHz, CDCl_3_) δ: 7.62 (d, *J* = 9.6 Hz, 1H, CH); 7.57 (d, *J* = 16.0 Hz, 1H, CH=CH); 7.38 (d, *J* = 8.8 Hz, 1H, CH); 6.87 (dd, *J* = 8.8, 2.2 Hz, 1H, CH); 6.76 (d, *J* = 2.2 Hz, 1H, CH); 6.74 (s, 2H, CH); 6.33 (d, *J* = 16.0 Hz, 1H, CH=CH); 6.25 (d, *J* = 9.6 Hz, 1H, CH); 4.67 (s, 2H, OCH_2_); 4.22–4.15 (m, 4H, OCH_2_); 3.87 (s, 6H, OCH_3_); 3.86 (s, 3H, OCH_3_); 2.35–2.29 (m, 4H, NCH_2_); 2.20 (s, 3H, NCH_3_); 1.75–1.63 (m, 4H, CH_2_); 1.55–1.30 (m, 8H, CH_2_) ppm. ^13^C-NMR (100 MHz, CDCl_3_) δ: 167.99 (C); 167.01 (C); 160.86 (C); 160.82 (C); 155.66 (C); 153.43 (C); 144.61 (CH); 143.20 (CH); 140.21 (C); 129.93 (C); 128.97 (CH); 117.44 (CH); 113.76 (CH); 113.32 (C); 112.85 (CH); 105.24 (CH); 101.70 (CH); 65.66 (CH_2_); 65.34 (CH_2_); 64.54 (CH_2_); 60.95 (OCH_3_); 57.64 (CH_2_); 57.54 (CH_2_); 56.16 (OCH_3_); 42.15 (NCH_3_); 28.70 (CH_2_); 28.45 (CH_2_); 26.88 (CH_2_); 26.80 (CH_2_); 23.97 (CH_2_); 23.77 (CH_2_) ppm. ESI-HRMS (*m*/*z*) calculated for [M + H]^+^ ion species C_34_H_44_NO_10_ = 626.2960, found 626.2951.

*(E)-6-(methyl(3-(2-((2-oxo-2H-chromen-7-yl)oxy)acetoxy)propyl)amino)hexyl 3-(3,4,5-trimethoxyphenyl)acrylate***5**. From **39** [38]. Oil. Chromatographic eluent: CH_2_Cl_2_/CH_3_OH 96:4. Yield: 80%. ^1^H-NMR (400 MHz, CDCl_3_) δ: 7.61 (d, *J* = 9.6 Hz, 1H, CH); 7.56 (d, *J* = 16.0 Hz, 1H, CH=CH); 7.36 (d, *J* = 8.4 Hz, 1H, CH); 6.85 (dd, *J* = 8.4, 2.2 Hz, 1H, CH); 6.74 (d, *J* = 2.2 Hz, 1H, CH); 6.72 (s, 2H, CH); 6.32 (d, *J* = 16.0 Hz, 1H, CH=CH); 6.24 (d, *J* = 9.6 Hz, 1H, CH,); 4.66 (s, 2H, OCH_2_); 4.24 (t, *J* = 6.4 Hz, 2H, OCH_2_); 4.16 (t, *J* = 6.4 Hz, 2H, OCH_2_); 3.85 (s, 6H, OCH_3_); 3.84 (s, 3H, OCH_3_); 2.35 (t, *J* = 7.2 Hz, 2H, NCH_2_); 2.28 (t, *J* = 7.6 Hz, 2H, NCH_2_); 2.16 (s, 3H, NCH_3_); 1.84–1.77 (m, 2H, CH_2_); 1.70–1.64 (m, 2H, CH_2_); 1.47–1.30 (m, 6H, CH_2_) ppm. ^13^C-NMR (100 MHz, CDCl_3_) δ: 167.96 (C); 167.01 (C); 160.84 (C); 160.79 (C); 155.64 (C); 153.41 (C); 144.57 (CH); 143.20 (CH); 140.06 (C); 129.93 (C); 128.98 (CH); 117.45 (CH); 113.73 (CH); 113.31 (C); 112.77 (CH); 105.21 (CH); 101.70 (CH); 65.31 (CH_2_); 64.55 (CH_2_); 64.15 (CH_2_); 60.93 (OCH_3_); 57.62 (CH_2_); 56.14 (OCH_3_); 53.91 (CH_2_); 42.01 (NCH_3_); 28.71 (CH_2_); 27.09 (CH_2_); 26.40 (CH_2_); 25.90 (CH_2_) ppm. ESI-HRMS (*m*/*z*) calculated for [M + H]^+^ ion species C_33_H_42_NO_10_ = 612.2803, found 612.2794.

*(E)-6-(methyl(4-(2-((2-oxo-2H-chromen-7-yl)oxy)acetoxy)butyl)amino)hexyl 3-(3,4,5-trimethoxyphenyl)acrylate***6**. From **40** [38]. Oil. Chromatographic eluent: CH_2_Cl_2_/CH_3_OH/NH_4_OH 97:3:0.3. Yield: 100%. ^1^H-NMR (400 MHz, CDCl_3_) δ: 7.61 (d, *J* = 9.6 Hz, 1H, CH); 7.55 (d, *J* = 16.0 Hz, 1H, CH=CH); 7.36 (d, *J* = 8.4 Hz, 1H, CH); 6.84 (dd, *J* = 8.4, 2.2 Hz, 1H, CH); 6.73 (d, *J* = 2.2 Hz, 1H, CH); 6.71 (s, 2H, CH); 6.31 (d, *J* = 16.0 Hz, 1H, CH=CH); 6.23 (d, *J* = 9.6 Hz, 1H, CH); 4.65 (s, 2H, OCH_2_); 4.21–4.14 (m, 4H, OCH_2_); 3.85 (s, 6H, OCH_3_); 3.84 (s, 3H, OCH_3_) 2.32–2.27 (m, 4H, NCH_2_); 2.16 (s, 3H, NCH_3_); 1.69–1.62 (m, 4H, CH_2_); 1.51–1.29 (m, 8H, CH_2_) ppm. ^13^C-NMR (100 MHz, CDCl_3_) δ: 167.98 (C); 166.98 (C); 160.85 (C); 160.77 (C); 155.59 (C); 153.38 (C); 144.54 (CH); 143.26 (CH); 139.99 (C); 129.91 (C); 129.00 (CH); 117.44 (CH); 113.66 (CH); 113.29 (C); 112.73 (CH); 105.16 (CH); 101.69 (CH); 65.50 (CH_2_); 65.28 (CH_2_); 64.53 (CH_2_); 60.92 (OCH_3_); 57.57 (CH_2_); 56.96 (CH_2_); 56.12 (OCH_3_); 41.88 (NCH_3_); 28.68 (CH_2_); 27.11 (CH_2_); 26.44 (CH_2_); 25.89 (CH_2_) ppm. ESI-HRMS (*m*/*z*) calculated for [M + H]^+^ ion species C_34_H_44_NO_10_ = 626.2960, found 626.2966.

*(E)-7-(methyl(2-(2-((2-oxo-2H-chromen-7-yl)oxy)acetoxy)ethyl)amino)heptyl 3-(3,4,5-trimethoxyphenyl)acrylate***7**. From **44** [38]. Oil. Chromatographic eluent: CH_2_Cl_2_/CH_3_OH 95:5. Yield: 10%. ^1^H-NMR (400 MHz, CDCl_3_) δ: 7.63 (d, *J* = 9.6 Hz, 1H, CH); 7.58 (d, *J* = 16.0 Hz, 1H, CH=CH); 7.38 (d, *J* = 8.8 Hz, 1H, CH); 6.88 (dd, *J* = 8.8, 2.2 Hz, 1H, CH); 6.78 (d, *J* = 2.2 Hz, 1H, CH); 6.74 (s, 2H, CH); 6.33 (d, *J* = 16.0 Hz, 1H, CH=CH); 6.27 (d, *J* = 9.6 Hz, 1H, CH); 4.70 (s, 2H, OCH_2_); 4.31 (t, *J* = 5.6 Hz, 2H, OCH_2_); 4.18 (t, *J* = 5.6 Hz, 2H, OCH_2_); 3.88 (s, 6H, OCH_3_); 3.87 (s, 3H, OCH_3_); 2.64 (t, *J* = 5.6 Hz 2H, NCH_2_); 2.38 (t, *J* = 7.2 Hz, 2H, NCH_2_); 2.26 (s, 3H, NCH_3_); 1.71–1.65 (m, 4H, CH_2_); 1.47–1.30 (m, 6H, CH_2_) ppm. ^13^C-NMR (100 MHz, CDCl_3_) δ: 167.93 (C); 167.05 (C); 160.89 (C); 160.74 (C); 153.43 (C); 144.64 (CH); 143.21 (CH); 129.93 (C); 129.03 (CH); 117.43 (CH); 113.80 (CH); 113.38 (C); 112.79 (CH); 105.22 (CH); 101.84 (CH); 65.35 (CH_2_); 65.26 (CH_2_); 64.52 (CH_2_); 60.97 (OCH_3_); 57.53 (CH_2_); 56.17 (OCH_3_); 54.99 (CH_2_); 52.50 (NCH_3_); 29.02 (CH_2_); 28.66 (CH_2_); 27.07 (CH_2_); 25.86 (CH_2_) ppm. ESI-HRMS (*m/z*) calculated for [M + H]^+^ ion species C_33_H_42_NO_10_ = 612.2803, found 612.2794.

*(E)-7-(methyl(7-(2-((2-oxo-2H-chromen-7-yl)oxy)acetoxy)heptyl)amino)heptyl 3-(3,4,5-trimethoxyphenyl)acrylate***8**. From **41 [35]**. Oil. Chromatographic eluent: CH_2_Cl_2_/CH_3_OH 95:5. Yield: 31%. ^1^H-NMR (400 MHz, CDCl_3_) δ: 7.61 (d, *J* = 9.6 Hz, 1H, CH); 7.56 (d, *J* = 16.0 Hz, 1H, CH=CH); 7.37 (d, *J* = 8.8 Hz, 1H, CH); 6.84 (dd, *J* = 8.8, 2.2 Hz, 1H, CH); 6.75 (d, *J* = 2.2 Hz, 1H, CH); 6.73 (s, 2H, CH); 6.33 (d, *J* = 16.0 Hz, 1H, CH=CH); 6.24 (d, *J* = 9.6 Hz, 1H, CH); 4.66 (s, 2H, OCH_2_); 4.20–4.15 (m, 4H, OCH_2_); 3.86 (s, 6H, OCH_3_); 3.85 (s, 3H, OCH_3_); 2.34–2.28 (m, 4H, NCH_2_); 2.20 (s, 3H, NCH_3_); 1.70–1.62 (m, 4H, CH_2_); 1.50–1.20 (m, 16H, CH_2_) ppm. ^13^C-NMR (100 MHz, CDCl_3_) δ: 168.00 (C); 167.03 (C); 160.87 (C); 160.82 (C); 155.65 (C); 153.41 (C); 144.54 (CH); 143.22 (CH); 140.06 (C); 129.94 (C); 128.97 (CH); 117.48 (CH); 113.72 (CH); 113.30 (C); 112.82 (CH); 105.21 (CH); 101.70 (CH); 65.73 (CH_2_); 65.33 (CH_2_); 64.63 (CH_2_); 60.94 (OCH_3_); 57.77 (CH_2_); 57.74 (CH_2_); 56.14 (OCH_3_); 42.15 (NCH_3_); 29.20 (CH_2_); 29.08 (CH_2_); 28.69 (CH_2_); 28.43 (CH_2_); 27.44 (CH_2_); 27.38 (CH_2_); 27.04 (CH_2_); 27.02 (CH_2_); 25.92 (CH_2_); 25.72 (CH_2_) ppm. ESI-HRMS (*m*/*z*) calculated for [M + H]^+^ ion species C_38_H_52_NO_10_ = 682.3586, found 682.3573.

*3-(methyl(5-(2-((2-oxo-2H-chromen-7-yl)oxy)acetoxy)pentyl)amino)propyl 3,4,5-trimethoxy benzoate***9**. From **42 [37]**. Oil. Chromatographic eluent: CH_2_Cl_2_/CH_3_OH/NH_4_OH 90:10:1. Yield: 86%. ^1^H-NMR (400 MHz, CDCl_3_) δ: 7.61 (d, *J* = 9.2 Hz, 1H, CH); 7.36 (d, *J* =8.4 Hz, 1H, CH); 7.25 (s, 2H, CH); 6.85 (dd, *J* = 8.4, 2.2 Hz, 1H, CH); 6.73 (d, *J* = 2.2 Hz, 1H, CH); 6.23 (d, *J* = 9.2 Hz, 1H, CH); 4.66 (s, 2H, OCH_2_); 4.33 (t, *J* = 6.4 Hz, 2H, OCH_2_); 4.16 (t, *J* = 6.4 Hz, 2H, OCH_2_); 3.86 (s, 9H, OCH_3_); 2.56 (t, *J* = 6.8 Hz, 2H, NCH_2_); 2.41 (t, *J* =7.2 Hz, 2H, NCH_2_); 2.29 (s, 3H, NCH_3_); 2.00–1.95 (m, 2H, CH_2_); 1.67–1.60 (m, 2H, CH_2_); 1.52–1.46 (m, 2H, CH_2_); 1.35–1.27 (m, 2H, CH_2_) ppm. ^13^C-NMR (100 MHz, CDCl_3_) δ: 168.01 (C); 166.15 (C); 160.88 (C); 160.80 (C); 155.61 (C); 152.91 (C); 143.27 (CH); 142.19 (C); 129.00 (CH); 125.24 (C); 113.69 (CH); 113.28 (C); 112.86 (CH); 106.78 (CH); 101.63 (CH); 65.50 (CH_2_); 65.30 (CH_2_); 63.30 (CH_2_); 60.90 (OCH_3_); 57.19 (CH_2_); 56.24 (OCH_3_); 53.96 (CH_2_); 41.70 (NCH_3_); 28.36 (CH_2_); 26.37 (CH_2_); 26.18 (CH_2_); 23.62 (CH_2_) ppm. ESI-HRMS (*m*/*z*) calculated for [M + H]^+^ ion species C_30_H_38_NO_10_ = 572.2490, found 572.2479.

*6-(methyl(3-(2-((2-oxo-2H-chromen-7-yl)oxy)acetoxy)propyl)amino)hexyl 3,4,5-trimethoxy benzoate***10**. From **33**. Oil. Chromatographic eluent: CH_2_Cl_2_/CH_3_OH/NH_4_OH 90:10:1. Yield: 83%. ^1^H-NMR (400 MHz, CDCl_3_) δ: 7.61 (d, *J* = 9.6 Hz, 1H, CH); 7.37 (d, *J* = 8.4 Hz, 1H, CH); 7.27 (s, 2H, CH); 6.85 (d, *J* = 8.4 Hz, 1H, CH); 6.75 (s, 1H, CH); 6.24 (d, *J* = 9.6 Hz, 1H, CH); 4.67 (s, 2H, OCH_2_); 4.29–4.23 (m, 4H, OCH_2_); 3.88 (s, 9H, OCH_3_); 2.40 (t, *J* = 7.2 Hz, 2H, NCH_2_); 2.33 (t, *J* = 7.2 Hz, 2H, NCH_2_); 2.20 (s, 3H, NCH_3_); 1.83–1.73 (m, 4H, CH_2_); 1.47–1.34 (m, 6H, CH_2_) ppm. ^13^C-NMR (100 MHz, CDCl_3_) δ: 167.92 (C); 166.18 (C); 160.77 (C); 155.60 (C); 152.87 (C); 143.18 (CH); 142.14 (C); 128.97 (CH); 125.45 (C); 113.69 (CH); 113.29 (C); 112.72 (CH); 106.80 (CH); 101.68 (CH); 65.29 (CH_2_); 65.09 (CH_2_); 63.99 (CH_2_); 60.85 (OCH_3_); 57.49 (CH_2_); 56.22 (OCH_3_); 53.85 (CH_2_); 41.81 (NCH_3_); 28.68 (CH_2_); 27.02 (CH_2_); 26.89 (CH_2_); 26.22 (CH_2_); 25.89 (CH_2_) ppm. ESI-HRMS (*m/z*) calculated for [M + H]^+^ ion species C_31_H_40_NO_10_ = 586.2647, found 586.2643.

*7-(methyl(2-(2-((2-oxo-2H-chromen-7-yl)oxy)acetoxy)ethyl)amino)heptyl 3,4,5-trimethoxy benzoate***11**. From **45 [38]**. Oil. Chromatographic eluent: CH_2_Cl_2_/CH_3_OH/NH_4_OH 93:7:0.3. Yield: 89%. ^1^H-NMR (400 MHz, CDCl_3_) δ: 7.58 (d, *J* = 9.6 Hz, 1H, CH); 7.33 (d, *J* = 8.4 Hz, 1H, CH); 7.24 (s, 2H, CH); 6.83 (d, *J* = 8.4 Hz, 1H, CH); 6.73 (s, 1H, CH); 6.20 (d, *J* = 9.6 Hz, 1H, CH); 4.66 (s, 2H, OCH_2_); 4.29 (t, *J* = 5.6 Hz, 2H, OCH_2_); 4.24 (t, *J* = 6.4 Hz, 2H, OCH_2_); 3.85 (s, 9H, OCH_3_); 2.67 (t, *J* = 5.2 Hz, 2H, NCH_2_); 2.40 (t, *J* =7.6 Hz, 2H, NCH_2_); 2.27 (s, 3H, NCH_3_); 1.73–1.69 (m, 2H, CH_2_); 1.45–1.26 (m, 8H, CH_2_) ppm. ^13^C-NMR (100 MHz, CDCl_3_) δ: 167.86 (C); 166.08 (C); 160.70 (C); 155.52 (C); 152.82 (C); 143.21 (CH); 142.07 (C); 128.96 (CH); 125.43 (C); 113.54 (CH); 113.23 (C); 112.60 (CH); 106.74 (CH); 101.72 (CH); 65.21 (CH_2_); 65.06 (CH_2_); 62.40 (CH_2_); 60.76 (OCH_3_); 57.53 (CH_2_); 56.15 (OCH_3_); 55.10 (CH_2_); 42.00 (NCH_3_); 29.01 (CH_2_); 28.58 (CH_2_); 27.08 (CH_2_); 26.46 (CH_2_); 25.84 (CH_2_) ppm. ESI-HRMS (*m*/*z*) calculated for [M + H]^+^ ion species C_31_H_40_NO_10_ = 586.2647, found 586.2638.

*3-(methyl(5-(2-((2-oxo-2H-chromen-7-yl)oxy)acetoxy)pentyl)amino)propyl anthracene-9-carboxylate***12**. From **43 [37]**. Oil. Chromatographic eluent: CH_2_Cl_2_/CH_3_OH 95:5. Yield: 81%. ^1^H-NMR (400 MHz, CDCl_3_) δ: 8.51 (s, 1H, CH); 8.05–8.00 (m, 4H, CH); 7.56–7.46 (m, 5H, CH); 7.30 (d, *J* = 8.8 Hz, 1H, CH); 6.82 (dd, *J* = 8.8, 2.2 Hz, 1H, CH); 6.73 (d, *J* = 2.2 Hz, 1H, CH); 6.23 (d, *J* = 9.2 Hz, 1H, CH); 4.68 (t, *J* = 6.4 Hz, 2H, OCH_2_); 4.64 (s, 2H, OCH_2_); 4.19 (t, *J* = 6.4 Hz, 2H, OCH_2_); 2.54 (t, *J* =7.2 Hz, 2H, NCH_2_); 2.35 (t, *J* = 7.2 Hz, 2H, NCH_2_); 2.25 (s, 3H, NCH_3_); 2.11–2.02 (m, 2H, CH_2_); 1.68–1.61 (m, 2H, CH_2_); 1.53–1.45 (m, 2H, CH_2_); 1.39–1.30 (m, 2H, CH_2_) ppm. ^13^C-NMR (100 MHz, CDCl_3_) δ: 169.60 (C); 167.99 (C); 160.87 (C); 160.77 (C); 155.59 (C); 143.19 (CH); 130.97 (C); 129.29 (CH); 128.93 (CH); 128.64 (CH); 128.38 (C); 127.98 (C); 126.96 (CH); 125.48 (CH); 124.97 (CH); 113.66 (CH); 113.24 (C); 112.74 (CH); 101.68 (CH); 65.55 (CH_2_); 65.31 (CH_2_); 64.04 (CH_2_); 57.40 (CH_2_); 54.13 (CH_2_); 41.89 (NCH_3_); 28.38 (CH_2_); 26.59 (CH_2_); 26.39 (CH_2_); 23.67 (CH_2_) ppm. ESI-HRMS (*m*/*z*) calculated for [M + H]^+^ ion species C_35_H_36_NO_7_ = 582.2486, found 582.2489.

*6-(methyl(3-(2-((2-oxo-2H-chromen-7-yl)oxy)acetoxy)propyl)amino)hexyl anthracene-9-carboxylate***13**. From **34**. Oil. Chromatographic eluent: CH_2_Cl_2_/CH_3_OH/NH_4_OH 90:10:1. Yield: 98%. ^1^H-NMR (400 MHz, CDCl_3_) δ: 8.38 (s, 1H, CH); 7.96 (d, *J* = 8.4 Hz, 2H, CH); 7.90 (d, *J* = 8.4 Hz, 2H, CH); 7.47–7.37 (m, 5H, CH); 7.16 (d, *J* = 8.8 Hz, 1H, CH); 6.70 (dd, *J* = 8.8, 2.2 Hz, 1H, CH); 6.64 (d, *J* = 2.2 Hz, 1H, CH); 6.10 (d, *J* = 9.6 Hz, 1H, CH); 4.57–4.52 (m, 4H, OCH_2_); 4.17 (t, *J* = 6.4 Hz, 2H, OCH_2_); 2.27 (t, *J* = 6.8 Hz, 2H, NCH_2_); 2.21 (t, *J* = 6.8 Hz, 2H, NCH_2_); 2.09 (s, 3H, NCH_3_); 1.85–1.69 (m, 4H, CH_2_); 1.46–1.35 (m, 4H, CH_2_); 1.33–1.26 (m, 2H, CH_2_) ppm. ^13^C-NMR (100 MHz, CDCl_3_) δ: 169.71 (C); 167.94 (C); 160.85 (C); 160.75 (C); 155.60 (C); 143.16 (CH); 142.69 (C); 141.90 (C); 130.98 (C); 129.22 (CH); 128.95 (CH); 128.63 (CH); 128.36 (C); 128.16 (C); 126.93 (CH); 125.47 (CH); 124.99 (CH); 113.70 (CH); 113.28 (C); 112.69 (CH); 101.70 (CH); 65.84 (CH_2_); 65.30 (CH_2_); 64.06 (CH_2_); 57.52 (CH_2_); 53.87 (CH_2_); 41.91 (NCH_3_); 28.75 (CH_2_); 27.04 (CH_2_); 26.95 (CH_2_); 26.30 (CH_2_); 26.04 (CH_2_) ppm. ESI-HRMS (*m*/*z*) calculated for [M + H]^+^ ion species C_36_H_38_NO_7_ = 596.2643, found 596.2652.

*7-(methyl(2-(2-((2-oxo-2H-chromen-7-yl)oxy)acetoxy)ethyl)amino)heptyl anthracene-9-carboxylate***14**. From **46 [38]**. Oil. Chromatographic eluent: CH_2_Cl_2_/CH_3_OH/NH_4_OH 96:4:0.4. Yield: 68%. ^1^H-NMR (400 MHz, CDCl_3_) δ: 8.48 (s, 1H, CH); 8.03–7.98 (m, 4H, CH); 7.54–7.44 (m, 5H, CH); 7.28 (d, *J* = 8.4 Hz, 1H, CH); 6.80 (dd, *J* = 8.4, 2.2 Hz, 1H, CH); 6.73 (d, *J* = 2.2 Hz, 1H, CH); 6.20 (d, *J* = 9.6 Hz, 1H, CH); 4.64 (s, 2H, OCH_2_); 4.60 (t, *J* = 6.8 Hz, 2H, OCH_2_); 4.29 (t, *J* = 5.6 Hz, 2H, OCH_2_); 2.62 (t, *J* = 5.6 Hz, 2H, NCH_2_); 2.36 (t, *J* = 7.2 Hz, 2H, NCH_2_); 2.24 (s, 3H, NCH_3_); 1.90–1.83 (m, 2H, CH_2_); 1.50–1.25 (m, 8H, CH_2_) ppm. ^13^C-NMR (100 MHz, CDCl_3_) δ: 169.71 (C); 167.94 (C); 160.85 (C); 160.73 (C); 155.59 (C); 143.15 (CH); 130.98 (C); 129.19 (CH); 128.91 (CH); 128.61 (CH); 128.35 (C); 126.90 (CH); 125.45 (CH); 124.99 (CH); 113.68 (CH); 113.26 (C); 112.71 (CH); 101.75 (CH); 65.87 (CH_2_); 65.29 (CH_2_); 63.11 (CH_2_); 57.91 (CH_2_); 55.48 (CH_2_); 42.49 (NCH_3_); 29.14 (CH_2_); 28.72 (CH_2_); 27.24 (CH_2_); 27.07 (CH_2_); 26.07 (CH_2_) ppm. ESI-HRMS (*m*/*z*) calculated for [M + H]^+^ ion species C_36_H_38_NO_7_ = 596.2643, found 596.2631.

#### 3.1.2. General Procedure for the Synthesis of Diester Compounds **15**–**28**

A 1 mmol portion of 4-sulfamoylbenzoic acid was transformed into the acyl chloride by reaction with SOCl_2_ (2 mmol) in 5 mL of CHCl_3_ (free of ethanol) at 60 °C for 5 h. The reaction mixture was cooled to room temperature, and the solvent was removed under reduced pressure; the mixture was then treated twice with cyclohexane and the solvent removed under reduced the pressure. The acyl chloride obtained was dissolved in CHCl_3_ (free of ethanol), and the suitable (hydroxyalkyl)methylaminoester **33**–**46** (1 eq) was added. The mixture was stirred for 17 h at room temperature and the solvent was removed under reduce pressure. Then CH_2_Cl_2_ was added and the organic layer was washed twice with a saturated solution of NaHCO_3_. After drying with Na_2_SO_4_, the solvent was removed under reduced pressure. The crude product was then purified by flash chromatography using the proper eluting system, yielding the desired compound as an oil.

All the compounds were transformed into the corresponding hydrochloride as a white solid. The salts were crystallized from abs. ethanol/petroleum ether.

*(E)-3-(methyl(3-((3-(3,4,5-trimethoxyphenyl)acryloyl)oxy)propyl)amino)propyl 4-sulfamoyl benzoate***15**. From (hydroxyalkyl)methylaminoester **35 [35]**. Oil. Chromatographic eluent: CH_2_Cl_2_/CH_3_OH/NH_4_OH 93:7:0.3. Yield: 63%. ^1^H-NMR (400 MHz, CDCl_3_) δ: 8.02 (d, *J* = 8.4 Hz, 2H, CH); 7.88 (d, *J* = 8.4 Hz, 2H, CH); 7.54 (d, *J* = 16.0 Hz, 1H, CH=CH); 6.69 (s, 2H, CH); 6.30 (d, *J* = 16.0 Hz, 1H, CH=CH); 4.35 (t, *J* = 6.4 Hz, 2H, OCH_2_); 4.22 (t, *J* = 6.0 Hz, 2H, OCH_2_); 3.83 (s, 6H, OCH_3_); 3.82 (s, 3H, OCH_3_); 2.51–2.46 (m, 4H, NCH_2_); 2.23 (s, 3H, NCH_3_); 1.95–1.81 (m, 4H, CH_2_) ppm. ^13^C-NMR (100 MHz, CDCl_3_) δ: 167.04 (C); 165.14 (C); 153.31 (C); 146.14 (C); 144.75 (CH); 139.89 (C); 133.72 (C); 130.10 (CH); 129.94 (C); 126.29 (CH); 117.29 (CH); 105.28 (CH); 64.04 (CH_2_); 62.70 (CH_2_); 60.95 (OCH_3_); 56.16 (OCH_3_); 54.00 (CH_2_); 53.69 (CH_2_); 41.99 (NCH_3_); 26.47 (CH_2_); 26.43 (CH_2_) ppm. ESI-HRMS (*m*/*z*) calculated for [M + H]^+^ ion species C_26_H_35_N_2_O_9_S = 551.2058, found 551.2050. Hydrochloride: low melting solid.

*(E)-5-(methyl(3-((3-(3,4,5-trimethoxyphenyl)acryloyl)oxy)propyl)amino)pentyl 4-sulfamoyl benzoate***16**. From **36 [37]**. Oil. Chromatographic eluent: CH_2_Cl_2_/CH_3_OH/NH_4_OH 93:7:0.3. Yield: 21%. ^1^H-NMR (400 MHz, CDCl_3_) δ: 8.09 (d, *J* = 8.4 Hz, 2H, CH); 7.95 (d, *J* = 8.4 Hz, 2H, CH); 7.58 (d, *J* = 16.0 Hz, 1H, CH=CH); 6.73 (s, 2H, CH); 6.33 (d, *J* = 16.0 Hz, 1H, CH=CH); 4.33 (t, *J* = 6.8 Hz, 2H, OCH_2_); 4.22 (t, *J* = 6.4 Hz, 2H, OCH_2_); 3.86 (s, 6H, OCH_3_); 3.85 (s, 3H, OCH_3_); 2.48 (t, *J* = 7.2 Hz, 2H, NCH_2_); 2.39 (t, *J* = 7.2 Hz, 2H, NCH_2_); 2.24 (s, 3H, NCH_3_); 1.89–1.74 (m, 4H, CH_2_); 1.59–1.41 (m, 4H, CH_2_) ppm. ^13^C-NMR (100 MHz, CDCl_3_) δ: 167.06 (C); 165.15 (C); 153.39 (C); 146.05 (C); 144.83 (CH); 140.09 (C); 133.97 (C); 130.17 (CH); 129.90 (C); 126.37 (CH); 117.25 (CH); 105.35 (CH); 65.67 (CH_2_); 62.88 (CH_2_); 60.94 (OCH_3_); 57.43 (CH_2_); 56.18 (OCH_3_); 53.93 (CH_2_); 41.99 (NCH_3_); 28.48 (CH_2_); 26.76 (CH_2_); 26.48 (CH_2_); 23.84 (CH_2_) ppm. ESI-HRMS (*m*/*z*) calculated for [M + H]^+^ ion species C_28_H_39_N_2_O_9_S = 579.2371, found 579.2380. Hydrochloride: mp 98–100 °C.

*(E)-3-(methyl(5-((3-(3,4,5-trimethoxyphenyl)acryloyl)oxy)pentyl)amino)propyl 4-sulfamoyl benzoate***17**. From **37 [37]**. Oil. Chromatographic eluent: CH_2_Cl_2_/CH_3_OH/NH_4_OH 93:7:0.3. Yield: 100%. ^1^H-NMR (400 MHz, CDCl_3_) δ: 8.10 (d, *J* = 8.4 Hz, 2H, CH); 7.96 (d, *J* = 8.4 Hz, 2H, CH); 7.58 (d, *J* = 16.0 Hz, 1H, CH=CH); 6.73 (s, 2H, CH); 6.34 (d, *J* = 16.0 Hz, 1H, CH=CH); 4.40 (t, *J* = 6.4 Hz, 2H, OCH_2_); 4.15 (t, *J* = 6.8 Hz, 2H, OCH_2_); 3.87 (s, 6H, OCH_3_); 3.86 (s, 3H, OCH_3_); 2.48 (t, *J* = 6.8 Hz, 2H, NCH_2_); 2.35 (t, *J* = 7.2 Hz, 2H, NCH_2_); 2.22 (s, 3H, NCH_3_); 1.96–1.90 (m, 2H, CH_2_); 1.71–1.64 (m, 2H, CH_2_); 1.51–1.40 (m, 4H, CH_2_) ppm. ^13^C-NMR (100 MHz, CDCl_3_) δ: 167.23 (C); 165.14 (C); 153.35 (C); 146.22 (C); 144.81 (CH); 139.97 (C); 133.79 (C); 130.14 (CH); 129.93 (C); 126.35 (CH); 117.33 (CH); 105.27 (CH); 64.53 (CH_2_); 64.06 (CH_2_); 60.94 (OCH_3_); 57.50 (CH_2_); 56.15 (OCH_3_); 53.75 (CH_2_); 42.14 (NCH_3_); 28.66 (CH_2_); 26.87 (CH_2_); 26.39 (CH_2_); 23.81 (CH_2_) ppm. ESI-HRMS (*m*/*z*) calculated for [M + H]^+^ ion species C_28_H_39_N_2_O_9_S = 579.2371, found 579.2364. Hydrochloride: mp 83–86 °C.

*(E)-5-(methyl(5-((3-(3,4,5-trimethoxyphenyl)acryloyl)oxy)pentyl)amino)pentyl 4-sulfamoyl benzoate***18**. From **38 [35]**. Oil. Chromatographic eluent: CH_2_Cl_2_/CH_3_OH/NH_4_OH 97:3:0.3. Yield: 19%. ^1^H-NMR (400 MHz, CDCl_3_) δ: 8.11 (d, *J* = 8.4 Hz, 2H, CH); 7.96 (d, *J* = 8.4 Hz, 2H, CH); 7.58 (d, *J* = 16.0 Hz, 1H, CH=CH); 6.74 (s, 2H, CH); 6.33 (d, *J* = 16.0 Hz, 1H, CH=CH); 4.33 (t, *J* = 6.4 Hz, 2H, OCH_2_); 4.17 (t, *J* = 6.8 Hz, 2H, OCH_2_); 3.87 (s, 6H, OCH_3_); 3.85 (s, 3H, OCH_3_); 2.40–2.32 (m, 4H, NCH_2_); 2.22 (s, 3H, NCH_3_); 1.83–1.66 (m, 4H, CH_2_); 1.58–1.37 (m, 8H, CH_2_) ppm. ^13^C-NMR (100 MHz, CDCl_3_) δ: 167.15 (C); 165.17 (C); 153.41 (C); 146.25 (C); 144.76 (CH); 140.14 (C); 133.94 (C); 130.17 (CH); 129.92 (C); 126.36 (CH); 117.36 (CH); 105.36 (CH); 65.79 (CH_2_); 65.63 (CH_2_); 64.53 (CH_2_); 60.92 (OCH_3_); 57.41 (CH_2_); 57.38 (CH_2_); 56.17 (OCH_3_); 42.05 (NCH_3_); 28.65 (CH_2_); 28.53 (CH_2_); 26.64 (CH_2_); 23.96 (CH_2_); 23.90 (CH_2_) ppm. ESI-HRMS (*m*/*z*) calculated for [M + H]^+^ ion species C_30_H_43_N_2_O_9_S = 607.2684, found 607.2672. Hydrochloride: mp 83–85 °C.

*(E)-3-(methyl(6-((3-(3,4,5-trimethoxyphenyl)acryloyl)oxy)hexyl)amino)propyl 4-sulfamoyl benzoate***19**. From **39 [38]**. Oil. Chromatographic eluent: CH_2_Cl_2_/CH_3_OH/NH_4_OH 90:10:1. Yield: 40%. ^1^H-NMR (400 MHz, CDCl_3_) δ: 8.07 (d, *J* = 8.4 Hz, 2H, CH); 7.94 (d, *J* = 8.4 Hz, 2H, CH); 7.57 (d, *J* = 16.0 Hz, 1H, CH=CH); 6.73 (s, 2H, CH); 6.33 (d, *J* = 16.0 Hz, 1H, CH=CH); 4.99 (bs, 2H, NH_2_); 4.35 (t, *J* = 6.4 Hz, 2H, OCH_2_); 4.12 (t, *J* = 6.8 Hz, 2H, OCH_2_); 3.85 (s, 6H, OCH_3_); 3.83 (s, 3H, OCH_3_); 2.49 (t, *J* = 7.2 Hz, 2H, NCH_2_); 2.35 (t, *J* = 7.2 Hz, 2H, NCH_2_); 2.22 (s, 3H, NCH_3_); 1.95–1.88 (m, 2H, CH_2_); 1.69–1.60 (m, 2H, CH_2_); 1.49–1.32 (m, 6H, CH_2_) ppm. ^13^C-NMR (100 MHz, CDCl_3_) δ: 167.28 (C); 165.16 (C); 153.35 (C); 146.38 (C); 144. 83 (CH); 139.98 (C); 133.70 (C); 130.15 (CH); 129.91 (C); 126.34 (CH); 117.31 (CH); 105.26 (CH); 64.63 (CH_2_); 63.96 (CH_2_); 60.93 (OCH_3_); 57.51 (CH_2_); 56.15 (OCH_3_); 53.69 (CH_2_); 42.00 (NCH_3_); 28.63 (CH_2_); 27.01 (CH_2_); 26.93 (CH_2_); 26.22 (CH_2_); 25.87 (CH_2_) ppm. ESI-HRMS (*m*/*z*) calculated for [M + H]^+^ ion species C_29_H_41_N_2_O_9_S = 593.2527, found 593.2522. Hydrochloride: mp 73–76 °C.

*(E)-4-(methyl(6-((3-(3,4,5-trimethoxyphenyl)acryloyl)oxy)hexyl)amino)butyl 4-sulfamoyl benzoate***20**. From **40 [38]**. Oil. Chromatographic eluent: CH_2_Cl_2_/CH_3_OH/NH_4_OH 93:7:0.3. Yield: 49%. ^1^H-NMR (400 MHz, CDCl_3_) δ: 8.06 (d, *J* = 8.8 Hz, 2H, CH); 7.92 (d, *J* = 8.8 Hz, 2H, CH); 7.57 (d, *J* = 16.0 Hz, 1H, CH=CH); 6.73 (s, 2H, CH); 6.33 (d, *J* = 16.0 Hz, 1H, CH=CH); 5.50 (bs, 2H, NH_2_); 4.32 (t, *J* = 6.4 Hz, 2H, OCH_2_); 4.15 (t, *J* = 6.8 Hz, 2H, OCH_2_); 3.85 (s, 6H, OCH_3_); 3.84 (s, 3H, OCH_3_) 2.40–2.31 (m, 4H, NCH_2_); 2.20 (s, 3H, NCH_3_); 1.79–1.72 (m, 2H, CH_2_); 1.70–1.55 (m, 4H, CH_2_); 1.50–1.30 (m, 6H, CH_2_) ppm. ^13^C-NMR (100 MHz, CDCl_3_) δ: 167.22 (C); 165.21 (C); 153.35 (C); 146.39 (C); 144.75 (CH); 139.95 (C); 133.72 (C); 130.15 (CH); 129.92 (C); 126.31 (CH); 117.36 (CH); 105.21 (CH); 65.53 (CH_2_); 64.61 (CH_2_); 60.94 (OCH_3_); 57.45 (CH_2_); 57.03 (CH_2_); 56.14 (OCH_3_); 42.05 (NCH_3_); 28.66 (CH_2_); 27.14 (CH_2_); 26.84 (CH_2_); 26.60 (CH_2_); 25.86 (CH_2_); 23.62 (CH_2_) ppm. ESI-HRMS (*m*/*z*) calculated for [M + H]^+^ ion species C_30_H_43_N_2_O_9_S = 607.2684, found 607.2683. Hydrochloride: mp 89–91 °C.

*(E)-2-(methyl(7-((3-(3,4,5-trimethoxyphenyl)acryloyl)oxy)heptyl)amino)ethyl 4-sulfamoyl benzoate***21**. From **44 [38]**. Oil. Chromatographic eluent: CH_2_Cl_2_/CH_3_OH/NH_4_OH 93:7:0.3. Yield: 45%. ^1^H-NMR (400 MHz, CDCl_3_) δ: 8.11 (d, *J* = 8.8 Hz, 2H, CH); 7.95 (d, J = 8.8 Hz, 2H, CH); 7.59 (d, *J* = 15.6 Hz, 1H, CH=CH); 6.75 (s, 2H, CH); 6.34 (d, *J* = 15.6 Hz, 1H, CH=CH); 4.43 (t, *J* = 5.6 Hz, 2H, OCH_2_); 4.14 (t, *J* = 6.4 Hz, 2H, OCH_2_); 3.87 (s, 6H, OCH_3_); 3.86 (s, 3H, OCH_3_); 2.76 (t, *J* = 5.6 Hz, 2H, NCH_2_); 2.43 (t, *J* = 7.2 Hz, 2H, NCH_2_); 2.31 (s, 3H, NCH_3_); 1.66–1.62 (m, 2H, CH_2_); 1.49–1.45 (m, 2H, CH_2_); 1.33–1.25 (m, 6H, CH_2_) ppm. ^13^C-NMR (100 MHz, CDCl_3_) δ: 167.37 (C); 165.14 (C); 153.40 (C); 146.19 (C); 144.92 (CH); 140.07 (C); 133.83 (C); 130.29 (CH); 129.90 (C); 126.36 (CH); 117.29 (CH); 105.29 (CH); 64.73 (CH_2_); 63.46 (CH_2_); 60.96 (OCH_3_); 57.80 (CH_2_); 56.17 (OCH_3_); 55.47 (CH_2_); 42.73 (NCH_3_); 29.13 (CH_2_); 28.66 (CH_2_); 27.20 (CH_2_); 27.17 (CH_2_); 25.92 (CH_2_) ppm. ESI-HRMS (*m*/*z*) calculated for [M + H]^+^ ion species C_29_H_41_N_2_O_9_S = 593.2527, found 593.2524. Hydrochloride: mp 70–73 °C.

*(E)-7-(methyl(7-((3-(3,4,5-trimethoxyphenyl)acryloyl)oxy)heptyl)amino)heptyl 4-sulfamoyl benzoate***22**. From **41 [35]**. Oil. Chromatographic eluent: CH_2_Cl_2_/CH_3_OH/NH_4_OH 93:7:0.3. Yield: 22%. ^1^H-NMR (400 MHz, CDCl_3_) δ: 8.15 (d, *J* = 8.4 Hz, 2H, CH); 8.00 (d, *J* = 8.4 Hz, 2H, CH); 7.61 (d, *J* = 15.6 Hz, 1H, CH=CH); 6.77 (s, 2H, CH); 6.37 (d, J = 15.6 Hz, 1H, CH=CH); 4.36 (t, *J* = 6.8 Hz, 2H, OCH_2_); 4.20 (t, *J* = 6.4 Hz, 2H, OCH_2_); 3.90 (s, 6H, OCH_3_); 3.89 (s, 3H, OCH_3_); 2.40–2.35 (m, 4H, NCH_2_); 2.26 (s, 3H, NCH_3_); 1.82–1.65 (m, 4H, CH_2_); 1.52–1.30 (m, 16H, CH_2_) ppm. ^13^C-NMR (100 MHz, CDCl_3_) δ: 167.13 (C); 165.18 (C); 153.42 (C); 146.07 (C); 144.64 (CH); 139.96 (C); 134.09 (C); 130.22 (CH); 129.96 (C); 126.42 (CH); 117.46 (CH); 105.29 (CH); 65.78 (CH_2_); 64.66 (CH_2_); 60.95 (OCH_3_); 57.57 (CH_2_); 56.17 (OCH_3_); 42.02 (NCH_3_); 29.14 (CH_2_); 28.67 (CH_2_); 28.53 (CH_2_); 27.37 (CH_2_); 27.36 (CH_2_); 26.77 (CH_2_); 25.95 (CH_2_); 25.89 (CH_2_) ppm. ESI-HRMS (*m*/*z*) calculated for [M + H]^+^ ion species C_34_H_51_N_2_O_9_S = 663.3310, found 663.3298. Hydrochloride: mp 68–70 °C.

*3-(methyl(5-((4-sulfamoylbenzoyl)oxy)pentyl)amino)propyl 3,4,5-trimethoxybenzoate***23**. From **42 [37]**. Oil. Chromatographic eluent: CH_2_Cl_2_/CH_3_OH/NH_4_OH 93:7:0.3. Yield: 22%. ^1^H-NMR (400 MHz, CDCl_3_) δ: 8.11 (d, *J* = 8.8 Hz, 2H, CH); 7.97 (d, *J* = 8.8 Hz, 2H, CH); 7.28 (s, 2H, CH); 4.37–4.32 (m, 4H, CH_2_); 3.90 (s, 9H, OCH_3_); 2.62 (t, *J* = 6.8 Hz, 2H, NCH_2_); 2.51 (t, *J* = 6.8 Hz, 2H, NCH_2_); 2.35 (s, 3H, NCH_3_); 2.05–1.98 (m, 2H, CH_2_); 1.83–1.76 (m, 2H, CH_2_); 1.65–1.58 (m, 2H, CH_2_); 1.51–1.43 (m, 2H, CH_2_) ppm. ^13^C-NMR (100 MHz, CDCl_3_) δ: 166.25 (C); 165.15 (C); 152.91 (C); 146.07 (C); 133.93 (C); 130.21 (CH); 126.41 (CH); 125.20 (C); 106.87 (CH); 65.56 (CH_2_); 63.28 (CH_2_); 60.92 (OCH_3_); 57.29 (CH_2_); 56.28 (OCH_3_); 54.00 (CH_2_); 41.74 (NCH_3_); 28.42 (CH_2_); 26.33 (CH_2_); 26.16 (CH_2_); 23.82 (CH_2_) ppm. ESI-HRMS (*m*/*z*) calculated for [M + H]^+^ ion species C_26_H_37_N_2_O_9_S = 553.2214, found 553.2218. Hydrochloride: mp 52–55 °C.

*6-(methyl(3-((4-sulfamoylbenzoyl)oxy)propyl)amino)hexyl 3,4,5-trimethoxybenzoate***24**. From **33**. Oil. Chromatographic eluent: CH_2_Cl_2_/CH_3_OH/NH_4_OH 93:7:0.3. Yield: 55%. ^1^H-NMR (400 MHz, CDCl_3_) δ: 8.01 (d, *J* = 8.4 Hz, 2H, CH); 7.88 (d, *J* = 8.4 Hz, 2H, CH); 7.21 (s, 2H, CH); 5.56 (bs, 2H, NH_2_); 4.31 (t, *J* = 6.0 Hz, 2H, OCH_2_); 4.19 (t, *J* = 6.8 Hz, 2H, OCH_2_); 3.82 (s, 6H, OCH_3_); 3.81 (s, 3H, OCH_3_); 2.45 (t, *J* = 7.2 Hz, 2H, NCH_2_); 2.31 (t, *J* = 7.2 Hz, 2H, NCH_2_); 2.18 (s, 3H, NCH_3_); 1.90–1.85 (m, 2H, CH_2_); 1.69–1.65 (m, 2H, CH_2_); 1.42–1.28 (m, 6H, CH_2_) ppm. ^13^C-NMR (100 MHz, CDCl_3_) δ: 166.39 (C); 165.13 (C); 152.86 (C); 146.37 (C); 142.11 (C); 133.70 (C); 130.13 (CH); 126.33 (CH); 125.41 (C); 106.85 (CH); 65.20 (CH_2_); 63.98 (CH_2_); 60.87 (OCH_3_); 57.50 (CH_2_); 56.25 (OCH_3_); 53.77 (CH_2_); 41.94 (NCH_3_); 28.64 (CH_2_); 27.03 (CH_2_); 26.89 (CH_2_); 26.23 (CH_2_); 25.90 (CH_2_) ppm. ESI-HRMS (*m*/*z*) calculated for [M + H]^+^ ion species C_27_H_39_N_2_O_9_S = 567.2371, found 567.2380. Hydrochloride: mp 53–56 °C.

*7-(methyl(2-((4-sulfamoylbenzoyl)oxy)ethyl)amino)heptyl 3,4,5-trimethoxybenzoate***25**. From **45 [38]**. Oil. Chromatographic eluent: CH_2_Cl_2_/CH_3_OH/NH_4_OH 93:7:0.3. Yield: 40%. ^1^H-NMR (400 MHz, CDCl_3_) δ: 8.08 (d, *J* = 8.4 Hz, 2H, CH); 7.93 (d, *J* = 8.4 Hz, 2H, CH); 7.27 (s, 2H, CH); 4.43 (t, *J* = 5.6 Hz, 2H, OCH_2_); 4.26 (t, *J* = 6.8 Hz, 2H, OCH_2_); 3.89 (s, 6H, OCH_3_); 3.87 (s, 3H, OCH_3_); 2.78 (t, *J* = 5.6 Hz, 2H, NCH_2_); 2.44 (t, *J* = 7.2 Hz, 2H, NCH_2_); 2.32 (s, 3H, NCH_3_); 1.74–1.71 (m, 2H, CH_2_); 1.50–1.46 (m, 2H, CH_2_); 1.40–1.30 (m, 6H, CH_2_) ppm. ^13^C-NMR (100 MHz, CDCl_3_) δ: 166.48 (C); 165.14 (C); 152.88 (C); 146.32 (C); 142.14 (C); 133.65 (C); 130.24 (CH); 126.31 (CH); 125.40 (C); 106.86 (CH); 65.29 (CH_2_); 63.35 (CH_2_); 60.88 (OCH_3_); 57.81 (CH_2_); 56.26 (OCH_3_); 55.40 (CH_2_); 42.63 (NCH_3_); 29.12 (CH_2_); 28.63 (CH_2_); 27.19 (CH_2_); 27.02 (CH_2_); 25.93 (CH_2_) ppm. ESI-HRMS (*m*/*z*) calculated for [M + H]^+^ ion species C_27_H_39_N_2_O_9_S = 567.2371, found 567.2368. Hydrochloride: mp 60–63 °C.

*3-(methyl(5-((4-sulfamoylbenzoyl)oxy)pentyl)amino)propyl anthracene-9-carboxylate***26**. From **43 [37]**. Oil. Chromatographic eluent: CH_2_Cl_2_/CH_3_OH/NH_4_OH 93:7:0.3. Yield: 41%. ^1^H-NMR (400 MHz, CDCl_3_) δ: 8.52 (s, 1H, CH); 8.10 (d, *J* = 8.4 Hz, 2H, CH); 8.02 (d, *J* = 8.4 Hz, 4H, CH); 7.93 (d, *J* = 8.4 Hz, 2H, CH); 7.55–7.47 (m, 4H, CH); 4.66 (t, *J* = 6.4 Hz, 2H, OCH_2_); 4.32 (t, *J* = 6.4 Hz, 2H, OCH_2_); 2.60 (t, *J* = 7.2 Hz, 2H, NCH_2_); 2.44 (t, *J* = 7.2 Hz, 2H, NCH_2_); 2.29 (s, 3H, NCH_3_); 2.12–2.05 (m, 2H, CH_2_); 1.79–1.72 (m, 2H, CH_2_); 1.60–1.40 (m, 4H, CH_2_) ppm. ^13^C-NMR (100 MHz, CDCl_3_) δ: 169.68 (C); 165.17 (C); 146.00 (C); 133.88 (C); 130.94 (C); 130.20 (CH); 129.39 (CH); 128.67 (CH); 128.35 (C); 127.79 (C); 127.05 (CH); 126.37 (CH); 125.52 (CH); 124.89 (CH); 65.56 (CH_2_); 63.95 (CH_2_); 57.32 (CH_2_); 53.98 (CH_2_); 41.73 (NCH_3_); 28.42 (CH_2_); 26.06 (CH_2_); 23.82 (CH_2_) ppm. ESI-HRMS (*m*/*z*) calculated for [M + H]^+^ ion species C_31_H_35_N_2_O_6_S = 563.2210, found 563.2211. Hydrochloride: mp 82–84 °C.

*6-(methyl(3-((4-sulfamoylbenzoyl)oxy)propyl)amino)hexyl anthracene-9-carboxylate***27**. From **34**. Oil. Chromatographic eluent: CH_2_Cl_2_/CH_3_OH/NH_4_OH 93:7:0.3. Yield: 29%. ^1^H-NMR (400 MHz, CDCl_3_) δ: 8.51 (s, 1H, CH); 8.08 (d, *J* = 8.4 Hz, 2H, CH); 8.01 (d, *J* = 8.4 Hz, 4H, CH); 7.93 (d, *J* = 8.4 Hz, 2H, CH); 7.55–7.46 (m, 4H, CH); 5.08 (bs, 2H, NH_2_); 4.59 (t, *J* = 6.8 Hz, 2H, OCH_2_); 4.37 (t, *J* = 6.4 Hz, 2H, OCH_2_); 2.51 (t, *J* = 7.2 Hz, 2H, NCH_2_); 2.38 (t, *J* = 7.2 Hz, 2H, NCH_2_); 2.24 (s, 3H, NCH_3_); 1.97–1.82 (m, 4H, CH_2_); 1.51–1.35 (m, 6H, CH_2_) ppm. ^13^C-NMR (100 MHz, CDCl_3_) δ: 169.85 (C); 165.09 (C); 146.22 (C); 142.61 (C); 141.80 (C); 133.63 (C); 130.96 (C); 130.18 (CH); 129.28 (CH); 128.64 (CH); 128.32 (C); 128.01 (C); 127.00 (CH); 126.37 (CH); 125.50 (CH); 124.91 (CH); 65.85 (CH_2_); 63.71 (CH_2_); 57.23 (CH_2_); 53.71 (CH_2_); 41.60 (NCH_3_); 28.62 (CH_2_); 26.86 (CH_2_); 26.39 (CH_2_); 25.92 (CH_2_); 25.84 (CH_2_) ppm. ESI-HRMS (*m*/*z*) calculated for [M + H]^+^ ion species C_32_H_37_N_2_O_6_S = 577.2367, found 577.2361. Hydrochloride: mp 81–84 °C.

*7-(methyl(2-((4-sulfamoylbenzoyl)oxy)ethyl)amino)heptyl anthracene-9-carboxylate***28**. From **46 [38]**. Oil. Chromatographic eluent: CH_2_Cl_2_/CH_3_OH/NH_4_OH 93:7:0.3. Yield: 33%. ^1^H-NMR (400 MHz, CDCl_3_) δ: 8.52 (s, 1H, CH); 8.11 (d, *J* = 8.0 Hz, 2H, CH); 8.01 (d, *J* = 8.0 Hz, 4H, CH); 7.93 (d, *J* = 8.0 Hz, 2H, CH); 7.56–7.47 (m, 4H, CH); 5.31 (bs, 2H, NH_2_); 4.60 (t, *J* = 6.8 Hz, 2H, OCH_2_); 4.46 (t, *J* = 5.6 Hz, 2H, OCH_2_); 2.80 (t, *J* = 5.6 Hz, 2H, NCH_2_); 2.47 (t, *J* = 7.2 Hz, 2H, NCH_2_); 2.35 (s, 3H, NCH_3_); 1.88–1.82 (m, 2H, CH_2_); 1.54–1.30 (m, 8H, CH_2_) ppm. ^13^C-NMR (100 MHz, CDCl_3_) δ: 169.93 (C); 165.08 (C); 146.27 (C); 133.47 (C); 130.96 (C); 130.28 (CH); 129.28 (CH); 128.64 (CH); 128.32 (C); 128.01 (C); 127.00 (CH); 126.31 (CH); 125.50 (CH); 124.92 (CH); 65.97 (CH_2_); 62.86 (CH_2_); 57.65 (CH_2_); 55.18 (CH_2_); 42.34 (NCH_3_); 29.03 (CH_2_); 28.64 (CH_2_); 27.10 (CH_2_); 26.53 (CH_2_); 26.00 (CH_2_) ppm. ESI-HRMS (*m*/*z*) calculated for [M + H]^+^ ion species C_32_H_37_N_2_O_6_S = 577.2367, found 577.2374. Hydrochloride: mp 71–73 °C.

### 3.2. Stability Tests

#### 3.2.1. Chemicals

Acetonitrile (Chromasolv), formic acid and ammonium formate (MS grade), NaCl, KCl, Na_2_HPO_4_ 2H_2_O, KH_2_PO_4_ (Reagent grade) and verapamil hydrochloride (analytical standard, used as an internal standard) were purchased by Sigma-Aldrich (Milan, Italy). MilliQ water 18 MΩ cm^−1^ was obtained from Millipore’s Simplicity system (Milan-Italy). 

Phosphate buffer solution (PBS) was prepared by adding 8.01 g L^−1^ of NaCl, 0.2 g L^−1^ of KCl, 1.78 g L^−1^ of Na_2_HPO_4_ 2H_2_O and 0.27 g L^−1^ of KH_2_PO_4_.

#### 3.2.2. Preparation of Samples

Each sample was prepared adding 10 μL of working solution 1 to 100 μL of the tested matrix (PBS) in micro centrifuge tubes. The obtained solutions correspond to 1 μM of analyte.

Each set of samples was incubated in triplicate at four different times, 0, 30, 60 and 120 min at 37 °C. Therefore, the degradation profile of each analyte was represented by a batch of 12 samples (4 incubation times × 3 replicates). After the incubation, the samples were added with 300 μL of ISTD solution and centrifuged (room temperature for 5 min at 10,000 rpm). The supernatants were transferred in auto sampler vials and dried under a gentle stream of nitrogen.

The dried samples were dissolved in 1.0 mL of 10 mM of formic acid in mQ water: acetonitrile 80:20 solution. The obtained sample solutions were analyzed by LC–MS/MS methods described in the Supplementary Material.

### 3.3. Biology and Enzymology

#### 3.3.1. Cell lines and Cultures

The K562 cell line is a highly undifferentiated erythroleukemia originally derived from a patient with chronic myelogenous leukemia [41]. The K562 leukemia cells and the P-gp expressing K562/DOX cells were obtained from Prof. J. P. Marie (Hopital Hotel-Dieu, Paris, France). These cells were cultured in RPMI 1640 medium with GlutaMAX I (GIBCO) medium supplemented with 10% fetal calf serum (FCS; GIBCO) at 37 °C in a humidified incubator with 5% CO_2_. To maintain the resistance, every month, resistant cells were cultured for three days with 400 nM doxorubicin. K562/DOX cells overexpress almost exclusively the membrane glycoprotein P-gp [42].

Human colon adenocarcinoma cell line (LoVo) was isolated from a metastatic nodule, and its MDR variant, LoVo/DOX, were obtained from LoVo (parental line) by exposure to increasing concentrations of doxorubicin and maintained in vitro in Ham’s F12 medium supplemented with 10% fetal bovine serum and vitamins (Life Technology, Monza, Italy) at 37 °C in a 5% CO_2_ atmosphere [47]. These two cell lines have been kindly offered by Professor D’Incalci of the Department of Oncology, IRCCS (Istituto di Ricerche Farmacologiche Mario Negri, Milan, Italy).

#### 3.3.2. Rhodamine-123 (Rhd 123) Uptake

The inhibition of P-gp activity was evaluated by measuring the uptake of the P-gp substrate rhodamine-123 (Rhd 123) in K562/DOX and LoVo/DOX cells, in the absence or in the presence of compounds by a flow cytometric test. Briefly, K562/DOX cells were sedimented and diluted to obtain a cell suspension at 5 × 10^5^ cells/mL in complete RPMI 1640 medium; LoVo/DOX cells suspension was obtained by incubating monolayer cell cultures with EDTA and trypsin, then cells were diluted to obtain a cell suspension at 5 × 10^5^ cells/mL in complete Ham’s F12. Cells were loaded with Rhd 123, 5.0 µM, for 30 min at 37 °C in a humidified atmosphere with 5% CO_2_ in the presence of the tested compounds at 3.0, 10 and 30 µM concentrations, or of the reference compound verapamil at 3.0 µM concentration. An aliquot of cells (control) was incubated with the fluorochrome in the absence of inhibitors. All compounds were added 15 min before Rhd 123.

At the end of the uptake, cells were sedimented, washed twice in ice-cold PBS, placed in PBS on ice, and kept in the dark until flow cytometric analysis. Samples were analyzed on a FACScantoflow cytometer (Becton Dickinson, San Jose CA, USA) equipped with two lasers at 488/633 nm and FACSDIVA software. The green fluorescence of Rhd 123 was collected by a 530-nm band pass filter and at least 10,000 events were acquired. 

The histograms were generated by program GraphPad Prism 5 (GraphPad Prism software, Inc., San Diego, CA, USA); the data were obtained from the ratio between the average fluorescence intensity of the samples preincubated with the compounds under study and subsequently exposed to rhodamine, and the fluorescence of the sample exposed only to the rhodamine.

#### 3.3.3. Intrinsic Cytotoxicity

The intrinsic toxicity of the compounds under study was determined by an MTT assay [43] after exposing the parental cells (K562 and LoVo) and resistant cells (K562/DOX and LoVo/DOX) to three concentrations (3, 10 and 30 µM) for 72 h. The percentage of growth compared to the untreated control was transformed into histograms with the GraphPad Prism 5 program (Graph Pad software, Adalta, Arezzo, Italy).

#### 3.3.4. CA Inhibition Assay

An SX.18MV-R Applied Photophysics (Oxford, UK) stopped-flow instrument was used to assay the catalytic/inhibition of various CA isozymes [44]. Phenol Red (at a concentration of 0.2 mM) was used as an indicator, working at the absorbance maximum of 557 nm, with 10 mM Hepes (pH 7.4) as a buffer and, 0.1 M Na_2_SO_4_ or NaClO_4_ (for maintaining constant the ionic strength; these anions are not inhibitory in the used concentration), following the CA-catalyzed CO_2_ hydration reaction for a period of 5–10 s. Saturated CO_2_ solutions in water at 25 °C were used as a substrate. Stock solutions of inhibitors were prepared at a concentration of 10 μM (in DMSO-water 1:1, *v*/*v*) and dilutions up to 0.01 nM done with the assay buffer mentioned above. At least 7 different inhibitor concentrations have been used for measuring the inhibition constant. Inhibitor and enzyme solutions were preincubated together for 10 min at room temperature prior to the assay, in order to allow for the formation of the E–I complex. Triplicate experiments were done for each inhibitor concentration, and the values reported throughout the paper were the mean of such results. The inhibition constants were obtained by non-linear least-squares methods using the Cheng–Prusoff equation, as reported earlier [29], and represent the mean from at least three different determinations. All CA isozymes used here were recombinant proteins obtained as reported earlier by our group [48,49].

#### 3.3.5. Reverse Transcription RT-PCR

The expression levels of MDR1 and CAXII were analyzed by quantitative PCR (qRT-PCR) using a RotorGene 3000 (Qiagen, Hilden, Germany) instrument. Primers were purchased from IDT (Germany). Of the total RNA 500 ng was retro-transcribed using iScript (Bio-Rad, Hercules, CA, USA) and amplified with specific primers: for MDR1, Fw CAGCTATTCGAAGAGTGGGCACAAAC and Rv GCCTCTGCATCAGCTGGACTGTTG. For CAXII, Fw CAAAGGCCAGGAAGCATTCGTCCC and Rv ATGTGTGTGCAGTACAGGGCTGTCTC. PCR amplification was carried out by SsoAdvancedTM Universal SYBR^®^ Green Supermix (Bio-Rad, USA) according to the manual’s instruction. In the present analysis 18s rRNA was confirmed to be stable and was used as the normalizer Fw CGGCTACCACATCCAAGGAA and Rv GTTATTTTTCGTCACTACCTCCCCGGG. The RT-qPCR was performed using the following procedure: 98 °C for 2 min, 40 cycles of 98 °C for 5 s and 60 °C for 10 s. The program was set to reveal the melting curve of each amplicon from 60 to 95 °C with a read every 0.5 °C.

#### 3.3.6. Flow cytometric P-Glycoprotein Expression

Flow cytometric analysis was carried out on leukemic cells and on cell suspensions (10^6^ cells/mL) of colon carcinoma cells obtained by incubating monolayer cell cultures with EDTA and trypsin. For the P-gp determination on the cell surface, the cells were incubated for 30 min at RT with MAb FITC conjugate CD243. After washing with ice-cold phosphate buffer saline (PBS, pH 7.2) with 1% bovine serum albumin (BSA, Sigma), cells were immediately analyzed. Dead cells were excluded from the analysis by gate on cells size before the acquisitions. Ten thousand events were acquired and fluorescence was analyzed with a FACScanto flow cytometer (Becton Dickinson) equipped with a 15 mW, 488 nm, air-cooled argon ion laser. The fluorescence emissions were collected through a 530 nm band-pass filter for fluorescein.

For quantitative evaluation of P-gp expression, the mean fluorescence channel (MFC) was calculated by FCS5 express software (De Novo software). P-gp content in arbitrary units was expressed as the ratio of the MFC of labeled resistant cells on the MFC of parental ones.

#### 3.3.7. Doxorubicin IC_50_ and IC_20_ Evaluation on K562/DOX and LoVo/DOX Cell Lines

In regards to the leukemic line, K562/DOX, the values of doxorubicin IC_50_ and IC_20_ were extrapolated from the cytotoxicity curves previously performed [38].

For the LoVo/DOX cell line, the doxorubicin growth inhibition was determined by the MTT assay and the IC_50_ and IC_20_ values were determined. Approximately 10^4^ cells/well were plated onto 96-well plates and allowed to attach for 24 h before treatment with a range of drug concentrations from 3 × 10^−11^ to 3 × 10^−5^ M for 3 days; each concentration was repeated four times. At the end of the incubation the cells were processed according to the MTT protocol [43] and the optical density of the chromogenic product was measured at 570 nm. IC_50_ and IC_20_ values were determined graphically from relative survival curves obtained by GraphPad Prism 5 software (GraphPad, San Diego, CA, USA).

The resistance index to doxorubicin of the two resistant cell lines was obtained from the ratio between the doxorubicin IC_50_ (µM) values on the resistant and parental lines. The resistance index was 73 for K562/DOX cells [38] (IC_50_ = 2.47 ± 0.25 µM and 0.034 ± 0.008 µM on K562/DOX and K562 cells, respectively) and 13.5 for LoVo/DOX cells (IC_50_ = 0.85 ± 0.084 µM and 0.063 ± 0.0066 µM on LoVo/DOX and LoVo cells, respectively).

#### 3.3.8. Enhancement of Doxorubicin Toxicity in the Presence of Compounds **2, 5, 7, 8, 21, 25** and **26**.

Doxorubicin was used at the concentration corresponding to the IC_20_ of the resistant lines, which were 0.5 µM and 0.3 µM for K562/DOX and LoVo/DOX cell lines, respectively. Compounds **2**, **5**, **7**, **8**, **21**, **25** and **26** were studied at two concentrations, 3 and 10 µM.

To evaluate the enhancement of doxorubicin toxicity in the presence of the tested compounds, the cells, in exponential growth phase, were seeded at 10^4^ cells/well and solutions of either compounds or doxorubicin, or a solution of doxorubicin in combination with the compounds, were added to the wells repeated in quadruplicate. Then the plates were incubated at 37 °C for 72 h in a humidified atmosphere with 5% CO_2_/95% air. The MTT working solution was then added and plates were further incubated for 3 h. Following incubation cells and formazan crystals were inspected microscopically. The supernatant was then carefully removed by slow aspiration and the formazan crystals were dissolved in 150 mL of acidified isopropanol solution. The absorbance of the solution was then read on an automated plate reader at a wavelength of 570 nm. The increase in the toxicity of doxorubicin was quantified by the ratio between the growth of the cell sample treated with IC_20_ of doxorubicin and that of the cell sample treated with IC_20_ of doxorubicin in combination with the various concentrations of the tested compounds.

## 4. Conclusions

In this work a new series of P-glycoprotein and CA XII inhibitor hybrids was synthesized and studied for their ability to reverse the P-gp mediated MDR in cancer cells, which overexpressed both the transmembrane P-gp and CA XII proteins. A dual P-gp and CA XII inhibitory activity could be useful for a synergistic antitumor mechanism with consequent selective inhibition of P-glycoprotein overexpressed in resistant cancer cells.

The synthesized compounds are characterized by the presence of both P-gp and CA XII binding moieties, such as a *N,N*-bis(alkanol)amine diester scaffold combined with a coumarin or benzene sulfonamide moieties endowed with a dual P-gp and CA XII inhibitory activity.

Many compounds showed multitarget activity against the two transmembrane proteins P-gp and CA XII. Derivatives bearing a coumarin residue, displayed high and selective hCA XII inhibitory activity and a moderate P-gp inhibitory activity on K562/DOX cells, which overexpressed only P-gp. Interestingly, some coumarin compounds showed a synergistic MDR reversal effect; indeed, they were more active in the P-gp inhibition on LoVo/DOX cells, which overexpressed both P-gp and CA XII, than on K562/DOX cells, which overexpressed only P-gp, in both rhodamine-123 uptake and doxorubicin cytotoxicity enhancement tests.

In particular, the coumarin derivatives **7** and **8**, which incorporate a (*E*)-3-(3,4,5-trimethoxyphenyl)vinyl moiety linked to a 7-methylenes spacer (*n* = 7, Ar = **a**, see Table 1) showed the best inhibitory profile, being more potent on LoVo/DOX than on K562/DOX cells both in the Rhd 123 uptake test and in the doxorubicin cytotoxicity enhancement assay. Moreover, when tested at 10 μM concentrations, these two compounds showed higher activity than verapamil in both tests on LoVo/DOX line. In fact, they were able to increase the Rhd 123 intracellular fluorescence intensity with FR = 12.60 and 9.90, respectively, vs. FR = 6.40 of verapamil, and to reduce the cell growth rate from 80% to 34.9% and 32.2%, respectively, vs. 51.0% of verapamil. These two compounds were very potent and selective for hCA XII with respect to the physiological relevant cytosolic isoenzymes hCA I and hCA II (hCA XII, Ki = 9.1 and 7.1 nM for **7** and **8**, respectively, and hCA I and hCA II, Ki = >10,000 nM).

Taking all these results into account, we discovered a new class of hybrid derivatives with an innovative synergistic inhibitory activity mechanism on P-glycoprotein and carbonic anhydrases. More work is needed to optimize the interaction of these molecules with both targets and to study their pharmacokinetic properties. Notably, compounds **7** and **8** can be considered valuable candidates as chemosensitizers able to overcome P-gp mediated MDR in cancer cells with a synergistic mechanism that make them potentially selective inhibitors of P-glycoprotein overexpressed in resistant cancer cells.

## Figures and Tables

**Table 1:**
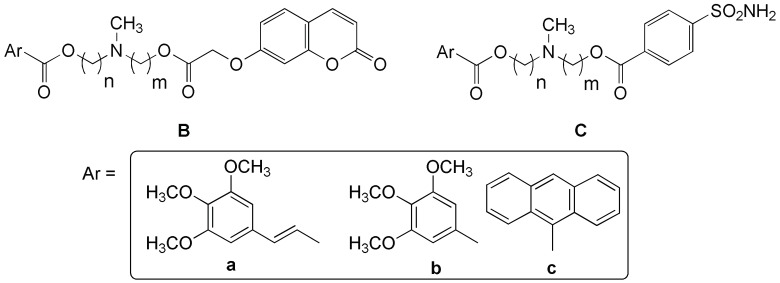
Rhd 123 uptake enhancement on K562/DOX cells and inhibitory activity on hCA I, II, IX and XII isoforms of compounds **1**–**28** and of the two reference compounds verapamil and acetazolamide (AAZ).

Compounds					FR ^a^		K_I_ (nM) ^b^			
N	n	m	Ar	Structure	3 µM	30 µM	hCA I	hCA II	hCA IX	hCA XII
**1**	3	3	a	B	1.44	4.76	>10,000	>10,000	142.5	9.3
**2**	3	5	a	B	1.57	5.81	>10,000	>10,000	47.4	8.9
**3**	5	3	a	B	1.00	2.58	>10,000	>10,000	54.6	48.8
**4**	5	5	a	B	1.99	5.45	>10,000	>10,000	26.6	18.9
**5**	6	3	a	B	3.67	5.85	>10,000	>10,000	26.7	6.4
**6**	6	4	a	B	1.39	1.98	>10,000	>10,000	40.2	36.4
**7**	7	2	a	B	2.08	4.48	>10,000	>10,000	29.7	9.1
**8**	7	7	a	B	2.33	4.15	>10,000	>10,000	24.2	7.1
**9**	3	5	b	B	1.00	3.95	>10,000	>10,000	133.3	9.2
**10**	6	3	b	B	1.16	2.18	>10,000	>10,000	103.1	8.9
**11**	7	2	b	B	1.11	1.58	>10,000	>10,000	97.1	8.3
**12**	3	5	c	B	1.00	2.53	>10,000	>10,000	150.8	45.7
**13**	6	3	c	B	1.43	2.45	>10,000	>10,000	82.7	9.1
**14**	7	2	c	B	1.25	4.22	>10,000	>10,000	125.8	42.9
**15**	3	3	a	C	1.00	2.30	55.1	151.7	178.4	91.0
**16**	3	5	a	C	1.08	1.83	447.2	554.8	210.6	94.3
**17**	5	3	a	C	1.13	3.90	56.6	203.7	215.3	33.1
**18**	5	5	a	C	1.00	4.99	62.6	424.8	226.5	62.6
**19**	6	3	a	C	1.06	3.23	509.6	8.4	39.7	58.6
**20**	6	4	a	C	1.01	2.58	84.5	14.0	33.9	41.7
**21**	7	2	a	C	1.30	3.70	533.2	48.9	68.4	35.2
**22**	7	7	a	C	2.85	3.20	73.7	445.0	171.9	96.7
**23**	3	5	b	C	1.00	1.66	284.4	127.5	26.6	63.7
**24**	6	3	b	C	1.42	2.88	83.5	8.9	25.3	57.6
**25**	7	2	b	C	1.44	3.50	42.7	90.2	117.0	8.0
**26**	3	5	c	C	2.10	4.45	338.7	81.2	241.2	23.4
**27**	6	3	c	C	1.08	2.03	616.7	422.4	28.2	31.3
**28**	7	2	c	C	1.45	4.25	237.9	475.5	20.6	23.5
**Ver**					3.30	4.71				
**AAZ**					1.00	1.00	250.0	12.0	25.0	5.7

^a^ Inhibition of the P-gp transport activity on K562/DOX cells expressed as FR that is the ratio between the average fluorescence intensity of rhodamine in the presence and in absence of modulators (FR = Rhd uptake + modulator/Rhd uptake − modulator). ^b^ Mean from 3 different assays, by a stopped flow technique (errors were in the range of ±5–10% of the reported values).

**Table 2:** Cells’ growth experiments performed on K562/DOX and LoVo/DOX cells in the presence of 0.5 µM (K562/DOX) or 0.3 µM (LoVo/DOX) concentrations of doxorubicin alone or in association with compounds **2**, **5**, **7**, **8**, **21**, **25** and **26** at 3 and 10 µM concentrations.

	Cell Growth (%) ^a^	
**Compounds**	K562/DOX cells	LoVo/DOX cells
doxo	80.0 ± 3.6	80.0 ± 2.5
doxo (IC_20_) + **2** (3 µM)	85.6 ± 4.7	82.7 ± 10.2
doxo (IC_20_) + **2** (10 µM)	65.0 ± 5.2	41.5 ± 7.3
doxo (IC_20_) + **5** (3 µM)	51.7 ± 3.0	58.0 ± 2.4
doxo (IC_20_) + **5** (10 µM)	25.1 ± 12.0	51.0 ± 1.9
doxo (IC_20_) + **7** (3 µM)	78.1 ± 4.9	61.0 ± 3.3
doxo (IC_20_) + **7** (10 µM)	48.0 ± 5.0	34.9 ± 8.1
doxo (IC_20_) + **8** (3 µM)	82.0 ± 5.9	68.0 ± 2.9
doxo (IC_20_) + **8** (10 µM)	49.0 ± 2.3	32.2 ± 10.7
doxo (IC_20_) + **21** (3 µM)	78.8 ± 10.2	82.0 ± 2.7
doxo (IC_20_) + **21** (10 µM)	68.7 ± 3.6	59.0 ± 3.3
doxo (IC_20_) + **25** (3 µM)	80.3 ± 5.9	81.7 ± 2.0
doxo (IC_20_) + **25** (10 µM)	76.0 ± 3.7	73.7 ± 0.015
doxo (IC_20_) + **26** (3 µM)	77.9 ± 6.1	82.0 ± 2.0
doxo (IC_20_) + **26** (10 µM)	74.0 ± 2.6	58.2 ± 5.9
doxo (IC_20_) + **Ver** (3 µM)	69.0 ± 0.03	54.0 ± 0.06
doxo (IC_20_) + **Ver** (10 µM)	62.0 ± 0.1	51.0 ± 0.09

^a^ Data are the mean ± SE of at least three determinations performed with quadruplicate cultures as described in Material and Methods.

## Supplementary Materials

The following are available online. Figure S1: mRNA expression level of MDR1 and CA XII genes, Figure S2: Fluorescence curves obtained with a FACScanto flow cytometer, Table S1: Elution gradient of mobile phase used for LC–MS/MS analyses, Table S2: MRM parameters, Table S3: Linear regression data, linearity coefficients, precision and LOD values for each analyte.
