# Supplementary material for: Dual P-Glycoprotein and CA XII Inhibitors: A New Strategy to Reverse the P-gp Mediated Multidrug Resistance (MDR) in Cancer Cells
Source: Molecules. 2020 Apr 10;25(7):1748. doi: 10.3390/molecules25071748 (PMC7181201; doi:10.3390/molecules25071748)
Supplement: Supplementary file 1 [file molecules-25-01748-s001.pdf]

Supplementary material

Dual P-glycoprotein and CA XII inhibitors: a new strategy to reverse the P-gp mediated multidrug resistance (MDR) in cancer cells

Elisabetta Teodori [1], Laura Braconi [1], Silvia Bua [1], Andrea Lapucci [2], Gianluca Bartolucci [1], Dina Manetti [1], Maria Novella Romanelli [1], Silvia Dei [1,*], Claudiu T. Supuran [1] and Marcella Coronnello [2]

[1] Department of Neuroscience, Psychology, Drug Research and Child's Health - Section of Pharmaceutical and Nutraceutical Sciences, University of Florence, via Ugo Schiff 6, 50019 Sesto Fiorentino (FI), Italy; elisabetta.teodori@unifi.it, laura.braconi@unifi.it, silvia.bua@unifi.it, gianluca.bartolucci@unifi.it, dina.manetti@unifi.it, novella.romanelli@unifi.it, silvia.dei@unifi.it, claudiu.supuran@unifi.it

[2] Department of Health Sciences - Section of Clinical Pharmacology and Oncology, University of Florence, Viale Pieraccini 6, 50139 Firenze, Italy; andrea.lapucci@unifi.it, marcella.coronnello@unifi.it

* Correspondence: silvia.dei@unifi.it

Table of Contents:

P-gp and CA XII expression in LoVo/DOX and K562/DOX cells by real-time PCR analysis S2

P-gp and CA XII expression in LoVo/DOX and K562/DOX cells using flow cytometric analysis S3

Chemical stability data of compounds **1-28** S4

Gene expression levels of P-gp and CA XII checked by real-time PCR analysis in K562 leukemic and LoVo colon carcinoma cells, both sensitive and resistant lines.

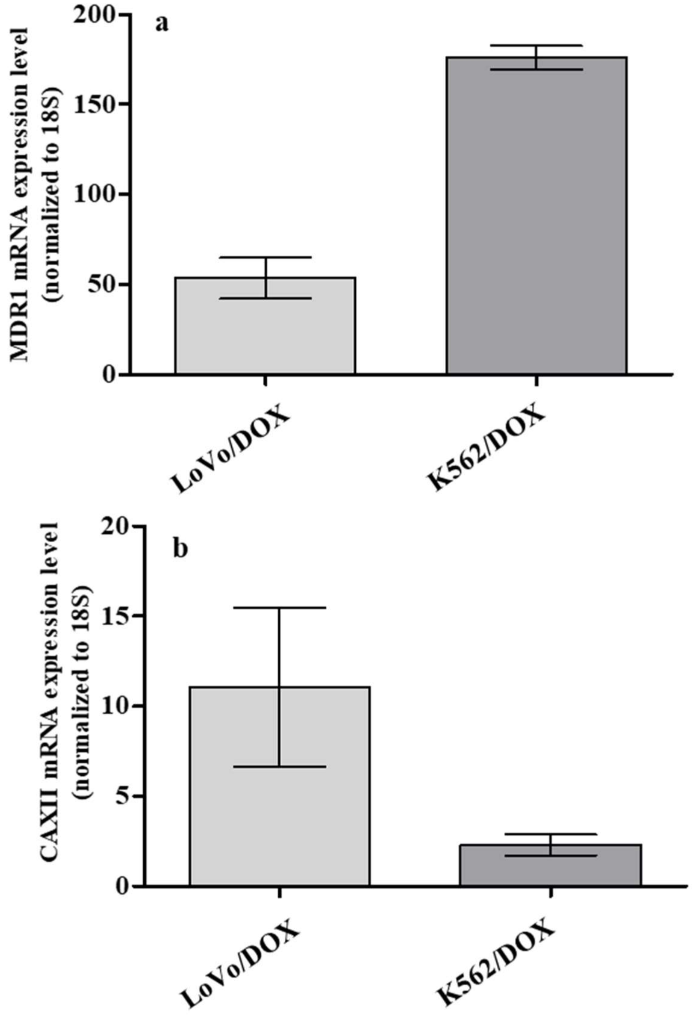

Figure S1. mRNA expression level of MDR1 (panel a) and CAXII (panel b) genes in the two doxorubicin resistant cell lines, K562/DOX and LoVo/DOX, due to P-gp overexpression. The data were obtained from the ratio between the mRNA expression levels on the resistant lines and the parental counterparts.

P-glycoprotein expression level of resistant K562/DOX and LoVo/DOX cells checked using flow cytometric analysis with the P-gp-specific monoclonal antibody Mab FITC conjugate CD243.

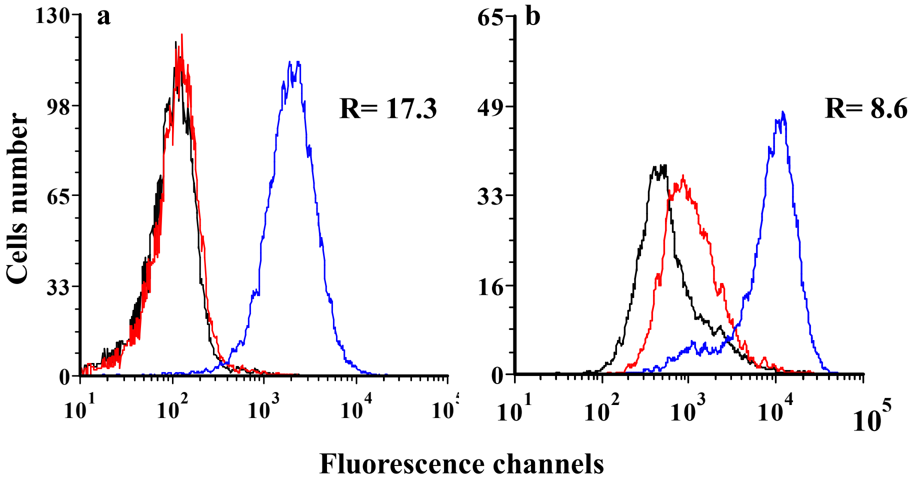

Figure S2. Fluorescence curves obtained with a FACScanto flow cytometer. R= ratio between the mean fluorescence intensity of resistant cells and parental cells. Panel a: K562 cells (red) and K562/DOX cells (blue); panel b: LoVo cells (red) and LoVo/DOX cells (blue); black curve: autofluorescence.

Chemical stability data

Instrumental

The LC-MS/MS analysis was carried out using a Varian 1200L triple quadrupole system (Palo Alto, CA, USA) equipped by two Prostar 210 pumps, a Prostar 410 autosampler and an Elettrospray Source (ESI) operating in positive ions mode. Raw-data were collected and processed by Varian Workstation Vers. 6.8 software. G-Therm 015 thermostatic oven was used to keep the samples at 37 °C during the degradation tests. Eppendorf microcentrifuge 5415D was employed to centrifuge plasma samples.

Standard solutions and calibration curves

Stock solutions of analytes and verapamil hydrochloride (ISTD) were prepared in acetonitrile at 1.0 mg mL^{-1} and stored at 4 °C. Working solutions of each analyte were freshly prepared by diluting stock solutions up to a concentration of 10 µM and 1 µM (working solution 1 and 2 respectively) in mQ water: acetonitrile 80:20 (v/v) solution. The ISTD working solution was prepared in acetonitrile at 60 ng mL^{-1} (ISTD solution).

A six levels calibration curve was prepared by adding proper volumes of working solution of each analyte to 300 µL of ISTD solution. The obtained solutions were dried under a gentle nitrogen stream and dissolved in 1.0 mL of 10 mM of formic acid in mQ water: acetonitrile 70:30 (v/v) solution. Final concentrations of calibration levels were: 0, 0.05, 0.10, 0.20, 0.50, 0.75 and 1.00 µM of analyte in the sample.

All calibration levels were analyzed six times by the appropriate LC-MS/MS method.

LC-MS/MS method

The chromatographic parameters employed to analyze the samples were tuned to minimize the run time and were reported as follows:

- column, Luna C18 length = 20 mm; internal diameter= 2 mm; particle size = 3 µm purchased from Phenomenex (Bologna, Italy)
- acidic mobile phase, composed by 5 mM of ammonium formate and 10 mM of formic acid in mQ water: acetonitrile 90:10 (v/v) solution (solvent A), 10 mM of ammonium formate and 5 mM of formic acid in mQ water: acetonitrile 10:90 (v/v) solution (solvent B).
- flow rate and the injection volume were 0.25 mL min^{-1} and 5 µL respectively.

The elution gradient is shown in Table S1.

The analyses were acquired in product ion scan, resonant excitation mode, parameters are reported in Table S2, using Nitrogen as collision gas at 3.0 mTorr.

Table S1: Elution gradient of mobile phase used for LC-MS/MS analyses.

Time (min)	A (%)
0.00	90
4.00	10
6.00	10
6.01	90
8.00	90

Table S2: MRM parameters.

Compounds	Precursor ion (m/z)	Quantitation ion (m/z) [CE (V)]	Qualification ion (m/z) [CE (V)]
**ISTD**	455	165 [30]	303 [30]
**1**	570	221 [40]	279 [40]
**2**	598	221 [40]	279 [35]
**3**	598	221 [40]	193 [50]
**4**	626	221 [45]	388 [45]
**5**	612	221 [45]	193 [45]
**6**	626	221 [40]	406 [40]
**7**	612	221 [45]	193 [45]
**8**	682	221 [50]	462 [50]
**9**	572	195 [45]	253 [40]
**10**	586	195 [45]	295 [40]
**11**	586	195 [45]	309 [40]
**12**	582	205 [35]	177 [50]
**13**	596	205 [40]	177 [50]
**14**	596	205 [40]	177 [50]
**15**	551	221 [35]	193 [50]
**16**	579	221 [35]	193 [50]
**17**	579	221 [40]	341 [40]
**18**	607	221 [40]	369 [40]
**19**	593	221 [40]	242 [45]
**20**	607	221 [40]	406 [40]
**21**	593	221 [45]	228 [40]
**22**	663	221 [50]	341 [50]
**23**	553	195 [45]	341 [35]
**24**	567	195 [45]	242 [45]
**25**	567	228 [40]	366 [35]
**26**	563	205 [35]	177 [50]
**27**	577	205 [45]	242 [45]
**28**	577	205 [40]	228 [45]

Linearity and LOD

Calibration curves of analytes were obtained by plotting the peak area ratios (PAR), between quantitation ions of analyte and ISTD, versus the nominal concentration of the calibration solution. A linear regression analysis was applied to obtain the best fitting function between the calibration points.

The precision was evaluated through the relative standard deviation (RSD%) of the quantitative data of the replicate analysis of highest level of calibration curves.

In order to obtain reliable LOD values, the standard deviation of response and slope approach was employed. The estimated standard deviations of responses were obtained by the standard deviation of y-intercepts (SDY-I) of regression lines. The obtained linear regressions, the linearity coefficients, precision and the estimated LOD values for each analyte are reported in Table S3.

Table S3: Linear regression data, linearity coefficients, precision and LOD values for each analyte.

Compounds	Slope (PAR/μM)	Intercept (PAR)	R^2	Precision (RSD)	LOD (μM)
**1**	0.719	0.007	0.999	4.70%	0.08
**2**	2.087	0.005	0.999	6.50%	0.05
**3**	2.101	0.006	0.999	6.30%	0.06
**4**	1.447	0.005	0.999	5.10%	0.09
**5**	3.350	0.011	0.998	1.90%	0.07
**6**	3.410	0.015	0.998	2.00%	0.06
**7**	3.280	0.013	0.998	1.80%	0.06
**8**	2.607	-0.011	0.999	1.40%	0.05
**9**	0.907	-0.004	0.999	4.50%	0.05
**10**	1.005	-0.001	0.999	3.90%	0.06
**11**	0.977	-0.003	0.998	4.10%	0.05
**12**	4.834	-0.011	0.999	0.30%	0.07
**13**	4.523	0.001	0.999	0.60%	0.05
**14**	4.689	-0.009	0.998	0.70%	0.06
**15**	2.138	-0.010	0.997	0.80%	0.08
**16**	6.177	0.001	0.998	0.80%	0.06
**17**	0.572	0.007	0.999	0.50%	0.08
**18**	1.374	-0.005	0.997	2.60%	0.05
**19**	0.687	0.007	0.999	1.90%	0.08
**20**	0.931	0.013	0.999	3.00%	0.05
**21**	0.191	0.009	0.999	1.60%	0.04
**22**	0.890	0.003	0.999	2.80%	0.07
**23**	1.063	0.002	0.999	5.00%	0.07
**24**	0.495	0.012	0.999	2.80%	0.09
**25**	0.496	-0.008	0.999	1.90%	0.05
**26**	0.849	-0.003	0.996	1.20%	0.05
**27**	1.206	-0.005	0.996	2.00%	0.05
**28**	0.350	0.007	0.999	1.10%	0.06
